# 3D Cellular Solar Crystallizer for Stable and Ultra‐Efficient High‐Salinity Wastewater Treatment

**DOI:** 10.1002/advs.202305313

**Published:** 2023-12-01

**Authors:** Can Wang, Hanchao Zhang, Zhanxiao Kang, Jintu Fan

**Affiliations:** ^1^ Research Centre of Textiles for Future Fashion, School of Fashion and Textiles The Hong Kong Polytechnic University Kowloon Hong Kong 999077 China

**Keywords:** high‐salinity brine treatment, interfacial solar crystallizers, real seawater, stable evaporation, zero liquid discharge

## Abstract

Recent developed interfacial solar brine crystallizers, which employ solar‐driven water evaporation for salts crystallization from the near‐saturation brine to achieve zero liquid discharge (ZLD) brine treatment, are promising due to their excellent energy efficiency and sustainability. However, most existing interfacial solar crystallizers are only tested using NaCl solution and failed to maintain high evaporation capability when treating real seawater due to the scaling problem caused by the crystallization of high‐valent cations. Herein, an artificial tree solar crystallizer (ATSC) with a multi‐branched and interconnected open‐cell cellular structure that significantly increased evaporation surface is rationally designed, achieving an ultra‐high evaporation rate (2.30 kg m^−2^ h^−1^ during 2 h exposure) and high energy efficiency (128%) in concentrated real seawater. The unit cell design of ATSC promoted salt crystallization on the outer frame rather than the inner voids, ensuring that salt crystallization does not affect the continuous transport of brine through the pores inside the unit cell, thus ATSC can maintain a stable evaporation rate of 1.94 kg m^−2^ h^−1^ on average in concentrated seawater for 80 h continuous exposure. The design concept of ATSC represents a major step forward toward ZLD treatment of high‐salinity brine in many industrial processes is believed.

## Graphical abstract

A rationally designed artificial tree solar crystallizer with multi‐branched and interconnected open‐cell cellular structures, which can maintain an ultra‐high and stable brine evaporation performance over a prolonged exposure (1.94 kg m^−2^ h^−1^ on average in concentrated real seawater for 80 h exposure), due to salt crystallization on the outer frame rather than the inner voids, ensuring continuous transport of brine.

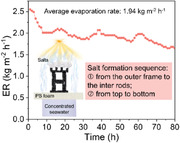

## 1 Introduction

In many industries, for example, seawater desalination, mining, petrochemical, and textile dyeing, high‐salinity wastewater is produced every day.^[^ ^1^, ^2^, ^3^, ^4^, ^5^, ^6^ ^]^ The daily discharge of high‐salinity wastewater from a plant may range from 0.1–10 000 m^3^, depending on the industrial process.^[^ ^7^, ^8^, ^9^ ^]^ Due to the cost and treatment difficulty, these brines are often discharged into aquatic environments without proper treatment, which causes a fatal impact on the aquatic ecosystems.^[^ ^10^, ^11^, ^12^, ^13^ ^]^ To address this issue, zero liquid discharge (ZLD) technology aimed at eliminating all waste liquid and producing solid salts as the only by‐product, is considered a promising strategy for maximizing resource recycling and minimizing wastewater discharge.^[^ ^14^, ^15^ ^]^ Traditional ZLD systems are composed of a concentration sub‐system and a crystallization sub‐system.^[^ ^16^ ^]^ The former concentrates the high‐salinity brine to near the saturation brine through reverse osmosis, electrodialysis, membrane distillation, and/or mechanical vapor compression concentrator.^[^ ^17^ ^]^ The latter then extract solid salts from the saturation brine using brine crystallizers or evaporation ponds.^[^ ^18^ ^]^ The crystallization process generally consumes huge amounts of electricity or fossil fuel with high capital costs.^[^ ^19^ ^]^ Therefore, it is crucial to develop a low‐cost, green and high‐performance brine crystallization process.

Interfacial solar brine crystallizers, which crystallize salts from the near‐saturation brines through local heating by solar‐thermal conversion near the liquid‐air interface, bring a new dimension to ZLD for their potential in efficiency and cost‐effectiveness.^[^ ^20^, ^21^, ^22^, ^23^, ^24^, ^25^ ^]^ Over the past few years, many efforts have been directed to advance the performance and stability of solar crystallizers for ZLD.^[^ ^26^, ^27^, ^28^, ^29^, ^30^ ^]^ In 2017, Finnerty et al.^[^ ^31^ ^]^ reported the possibility of achieving ZLD through salt accumulation by evaporation on “artificial leaves”. However, it was found, when treating 15 wt.% NaCl brine, the white salt layer formed on the surface could drastically decrease the evaporation rate to 0.5 kg m^−2^ h^−1^. To improve the evaporation performance, various solar brine crystallizers, such as the bio‐mimetic conical evaporator,^[^ ^32^ ^]^ 3D‐printed polylactic acid/carbon composites synthetic tree crystallizer,^[^ ^33^ ^]^ volcano‐like solar evaporator,^[^ ^34^ ^]^ and solar evaporators with localized salt crystallization^[^ ^35^ ^]^ were developed. Nevertheless, in most of these studies, NaCl solution was used as the surrogate for seawater, which however behave very differently from the real brine.^[^ ^36^, ^37^, ^38^, ^39^ ^]^ In 2021, Zhang et al.^[^ ^40^ ^]^ presented a novel design with the spatial isolation of salt crystallization from water evaporation and obtained a stable and high evaporation performance (1.61 kg m^−2^ h^−1^ for 24 h continuous evaporation) in 24 wt.% NaCl brine. However, their solar crystallizer lost its water evaporation capability after 20 h when treating real seawater brine. This is because loose NaCl crystals have less impact on the evaporation surface and brine‐wicking channels, but the multivalent ions in the real brine, especially Mg^2+^ and Ca^2+^, would generate the scales to block the pores of the wicking channels to restrain further evaporation.^[^ ^41^, ^42^ ^]^ The use of the crystallization inhibitor, nitrilotriacetic acid (NTA), could alleviate this salt‐clogged problem during the real brine evaporation process,^[^ ^29^ ^]^ but it would increase operating costs and bring secondary pollution to the ZLD system. Therefore, the development of solar crystallizers with stable and efficient real brine evaporation and salt accumulation is a remaining challenge and much needed for advanced ZLD technology.

In this work, we present a rationally designed artificial tree solar crystallizer (ATSC) for the real brine treatment with the ZLD goal. This ATSC is composed of multiple unit cells based on body‐centered cubic (BCC) with an added frame cellular microarchitecture, which could be fabricated by 3D printing. After coating with carbon black (CB) nanoparticles, the ATSC has high solar absorption and superhydrophilicity. Consequently, the cellular microarchitecture of ATSC could facilitate rapid wicking of brine through the multi‐branched and interconnected wicking channels from the “roots” to the “trunk” and then to the “leaves” of this structure. Besides, the 3D porous structure of ATSC hugely increased the available surface area for evaporation. The synergistic effect of ATSC make it an excellent water evaporator under solar radiation. We demonstrated that ATSC had an exceptionally high evaporation rate (2.30 kg m^−2^ h^−1^ over 2 h exposure) in concentrated real seawater under one sun radiation (1000 W m^−2^) with ultra‐high solar‐thermal conversion efficiency (128%). It was shown that the novel design of ATSC promoted salt crystallization on the outer frame of the unit cell, preventing the blocking of wicking channels for continuous brine evaporation. Additionally, the unit cell structure enhances the inhomogeneity of salt accumulation, resulting in the formed salt crust layer being irregular and highly porous, which ensured stable brine transport and light absorption even after prolonged salt accumulation in concentrated real seawater. As a result, ATSC maintained an exceptionally high and stable brine evaporation performance (average evaporation rate of 1.94 kg m^−2^ h^−1^) over 80 h in concentrated real seawater under one sun radiation without manual salt removal. The robust high performance and relatively low operating cost of ATSC is a major step forward toward the sustainable ZLD real brine treatment.

## 2 Results and Discussion

### 2.1 Design and Structure of the Tree‐Inspired Solar Crystallizer

**Figure 1:**
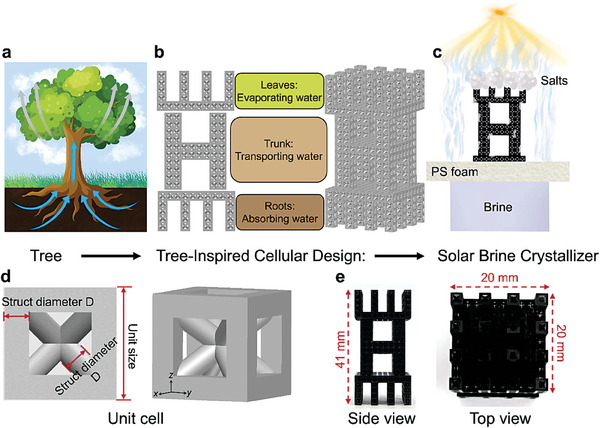
The design concept of the tree‐inspired cellular structure. a) and b) Natural tree and tree‐inspired cellular design for solar brine crystallizer, respectively. They share several key features: the bottom roots for absorbing water, the middle trunk for transporting water, and the upper leaves for absorbing sunlight and water evaporation. The cellular structure of ATSC can be designed and assembled by multiple unit cells. c) Schematic illustration of the ATSC under sunlight for salt crystallization from high‐salinity brine. d) Diagram of one unit cell in ATSC, which is body‐centered cubic (with frame). e) The side and top view of the ATSC.

In nature, trees have the intrinsic ability to use solar energy and groundwater to sustain themselves via the continuous transport of water and nutrients^[^ ^43^, ^44^ ^]^ from the bottom roots up to the trunk and top leaves through the vertically aligned channels (**Figure** **1**a), which inspired us to develop an ATSC and study its performance in the high‐salinity brine. Figure 1b presents a schematic diagram of the ATSC, which is assembled by roots, trunk, and leaves based on multiple unit cells, thus increasing the available evaporation surface by at least 3.4 times. This cellular microarchitecture of ATSC is scalable with multi‐branches and linked channels, thus enabling ATSC to have good water transmission and vapor escape properties, offering a novel approach toward ZLD brine treatment.

As illustrated in Figure 1c, to treat high‐salinity brine, we constructed an ATSC by one‐step 3D printing and dip coating in a carbon black (CB) solution. The raw material used for 3D printing is a resin consisting of acrylated monomer(s), photoinitiator(s), and urethane dimethacrylate. CB are widely used as solar‐absorbing material because of their excellent light absorption performance and low cost. The adhesion between CB nanoparticles and ATSC mainly involved van der Waals forces. The bonding between CB and ATSC was found to be sufficient during the photothermal process, as no additional CB shedding was found in the subsequent experimental process after the CB‐coated ATSC was rinsed with water immediately after coating. Figure S1 (Supporting Information) shows the ATSC shaking for five minutes in concentrated seawater. Clearly, there were no visible black particles in the concentrated seawater after shaking, indicating the CB was tightly bound to the solar crystallizer surface. The solar crystallizer was directly placed on top of a polystyrene (PS) foam with a low thermal conductivity (0.034–0.040 W m^−1^ K^−1^)^[^ ^45^ ^]^ to minimize the heat loss to the bulk brine. In addition to thermal insulation and light reflection, the PS foam provided buoyancy which enabled the ATSC to float on the brine while the root length of ATSC below the water was ≈2.80 mm (Figure S2, Supporting Information). The source brine was transported from the reservoir to the solar crystallizer by the root of the ATSC via capillary action. The evaporated water could be quickly compensated by continuous wicking of water through multi‐branched structures and interconnected channels. Furthermore, the design of ATSC allowed the brine to spread over the entire structure for evaporation. Over time, the salt accumulated on the crystallizer as water flew up the lattice and evaporated through.

The ATSC consisted of the unit cell of BCC with an added cubic frame (Figure 1d), including four body‐diagonals as inter rods and a cubic frame as the outer frame. Generally, the liquid capillary flow in simple tubes can be described by the Young–Laplace equation (ΔP  = 2γcos θ/*R* ) and Jurin's law.^[^ ^46^ ^]^ While it is complex in cellular open‐cell structures and is relevant to the liquid‐solid contact perimeter, surface tension, and contact angle.^[^ ^47^ ^]^ In our cells, this liquid‐solid boundary in a periodic manner with the liquid position in the cellular structure with local minima at the central node of the cell varied as a function of the struct diameter (D). Increasing the D reduces the effective pore size, the smaller capillary pores are necessary for a larger capillary rise. During the liquid‐wicking process within the cellular structure, the high capillary force and low flow resistance of the unit cell result in a higher overall liquid height. Furthermore, this type of unit cell has good mechanical strength and resistance to deformation,^[^ ^48^ ^]^ which is critical for collecting salts and reusing the crystallizer. This unit cell is a cubic structure with a unit size of 2.5 mm and a D varying from 0.4 to 0.6 mm. As D increases, the effective pore size decreases, leading to the rise in both capillary force and flow resistance of water transport. This cellular structure has numerous tetragonal pyramid cavities formed by the diagonal structs exposed to air, and air has extremely low thermal conductivity (≈0.023 W m^−1^ K^−1^)^[^ ^49^ ^]^ to minimize heat loss, thus enhancing localized heating. Figure 1e is the side and top views of ATSC, which shows the height and width of this structure were 41 and 20 mm, respectively.

### 2.2 Water Transport Performance

For efficient water evaporation, water transportation, light absorption, and thermal management properties of the solar brine crystallizers are the three key factors. To provide insight into the water transport performance of the ATSC composed of BCC (with added frame) unit cells, the wettability transition, cavity size change of the unit cell after coating with CB, and water‐wicking performance were investigated. To reveal the relationship between the water‐wicking performance and D of the unit cell, a series of columnar structures were prepared with increasing D of 0.40, 0.45, 0.50, 0.55, and 0.60 mm.

**Figure 2:**
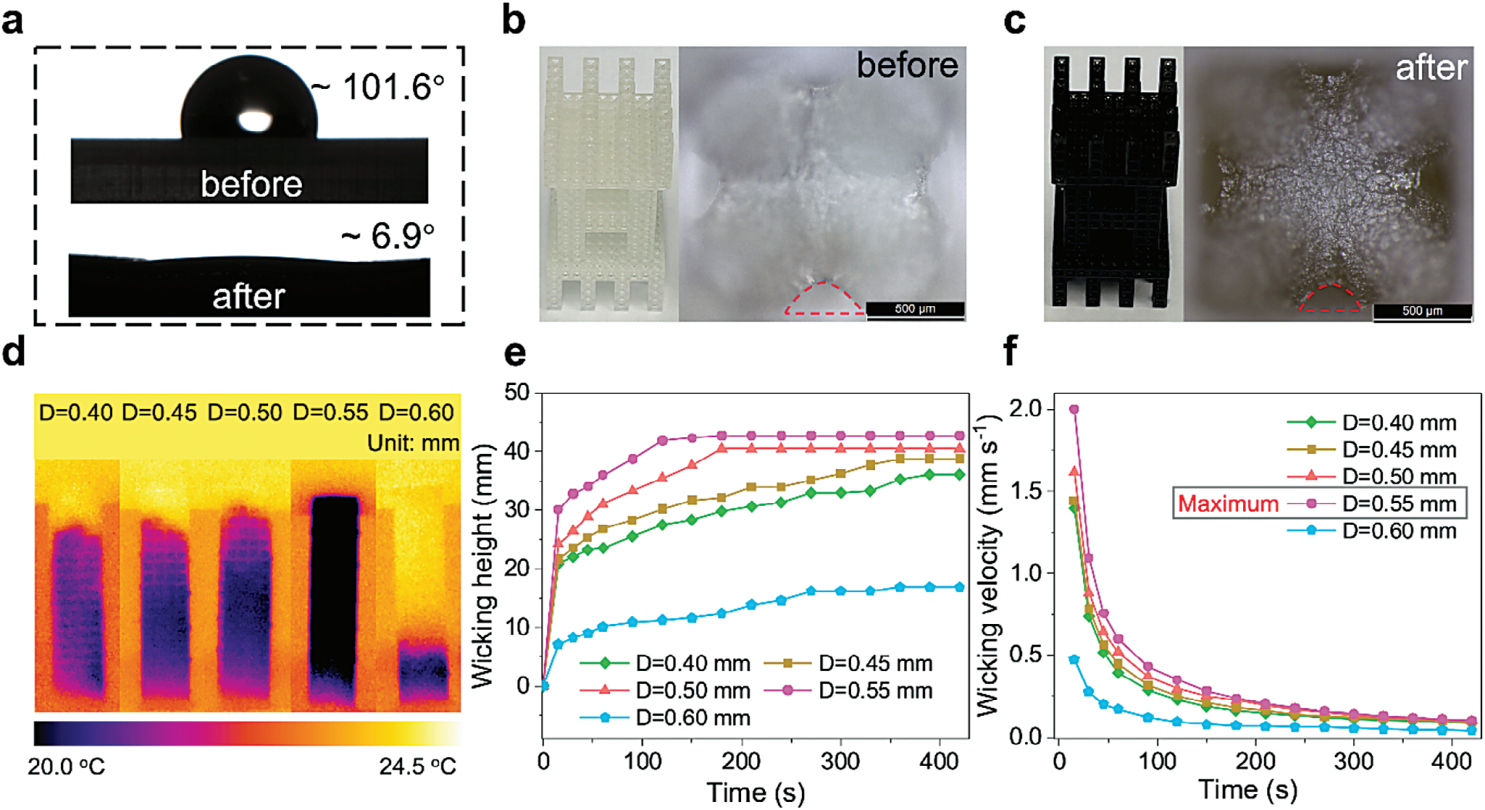
Water transport performance of the columnar structures with different D. a) The water contact angles of the ATSC before and after coating with CB. The optical images of the ATSC before b) and after c) coating with CB, demonstrate every void within the unit cell of ATSC reduced in size after coated. Anti‐gravity transport of water along the CB‐coated columnar structures composed with different D. d) The infrared (IR) images of the wicking height of five columnar structures. The corresponding wicking height e) and wicking velocity f) over time indicate that when D is equal to 0.55 mm, the columnar structure has the best capillary property.

As shown by X‐ray photoelectron spectroscopy (XPS) results, coated with CB increased the amounts of oxygen‐containing groups from 6.9% of O‐C = O to 7.3% of C‐OH (Figure S3a–c, Supporting Information). The water contact angles of ATSC changed from 101.6° to 6.9° after coating with CB (**Figure** **2**a), indicating that the surface of the CB‐coated ATSC was superhydrophilic. The scanning electron microscopy (SEM) images show that the surface of the ATSC changed to rather rough after coating with CB (Figure S3d,e, Supporting Information). Besides, Figure S3f (Supporting Information) indicates the size of CB nanoparticles is ≈30–50 nm. The color of the ATSC became black after coating with CB (Figure 2c), demonstrating that CB is successfully loading to the ATSC. As we can see from Figure 2b and c, the area marked with red dashed lines has been shrunk when every unit cell has been covered with CB. Figure S4 (Supporting Information) is the microscopy image of a CB‐coated unit cell, which shows every unit cell has six tetragonal pyramid cavities formed by the diagonal structs.

It was found that water can flow and wick quickly through the cellular columnar structure after coating with CB and that further increasing D resulted in a narrower effective pore size, which led to the wicking height first increasing and then decreasing (Figure 2d). When D was equal to 0.55 mm, the columnar structure reached the maximum wicking height of 42.7 mm in only 180 s (Figure 2e), showing the fast water upward transporting capability of the cellular columnar structure. Additionally, Figure 2f demonstrates that the wicking velocity increased as D increased first and then started to decrease when the D was larger than 0.55 mm, which was a similar trend to the wicking height. Although a higher capillary height can be obtained by increasing D due to the decreasing the effective pore size, the smaller pores also lead to a greater flow resistance which presents an insufficient permeability, resulting in a lower wicking velocity.^[^ ^50^ ^]^ Considering trade‐offs between capillary action and flow resistance, we choose 0.55 mm as the optimal D to compose the ATSC with continuous and rapid water transport capability and conduct subsequent evaporation experiments.

### 2.3 Light Absorption and Thermal Management Performance of ATSC

**Figure 3:**
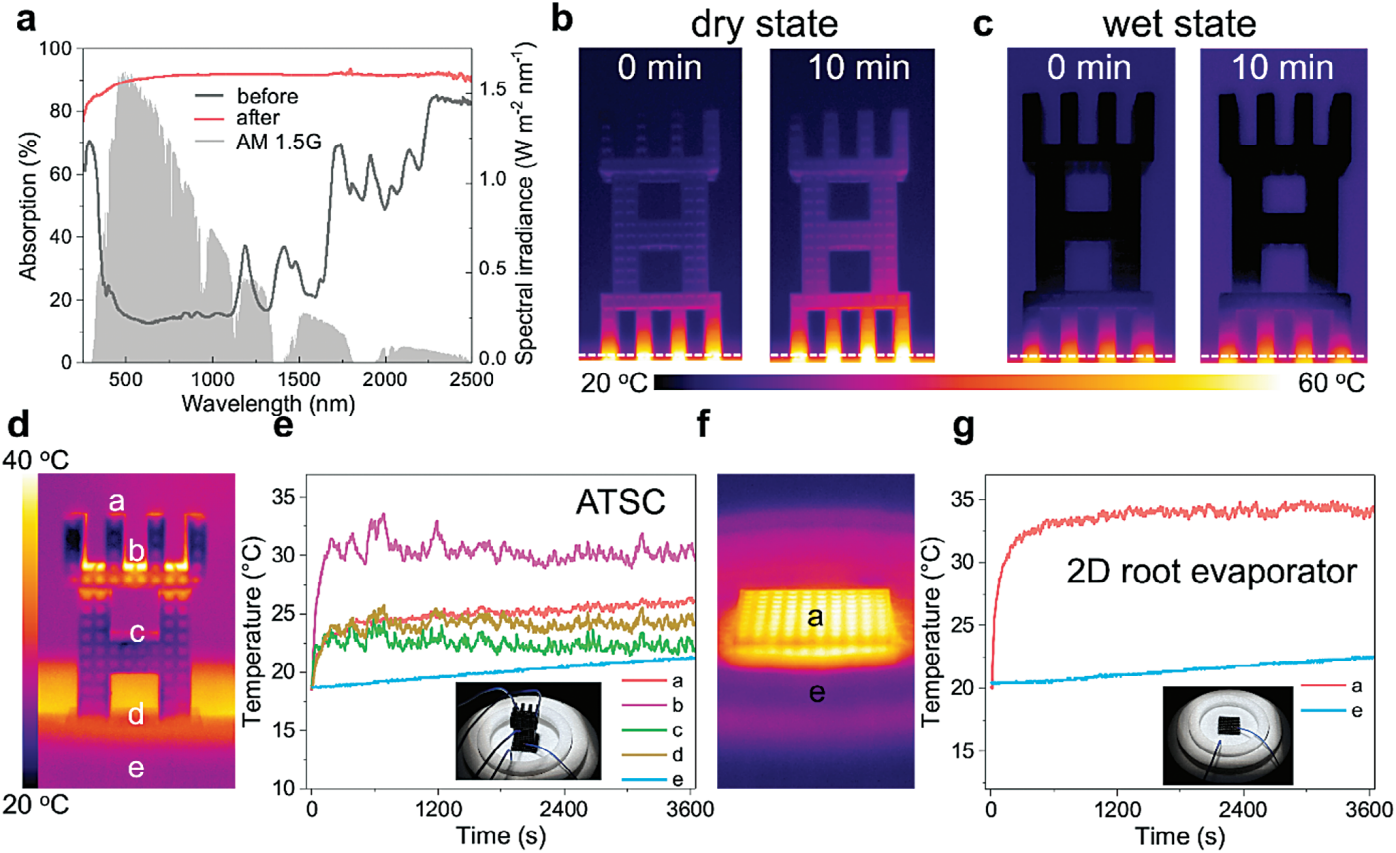
Light absorption and thermal management property of ATSC. a) The solar absorption spectra (250–2500 nm) of the ATSC before and after coating with CB, and standard AM 1.5G solar spectrum. The high solar absorption is contributed to both CB with high light absorbability and multiple light reflections of the cellular microarchitecture. Surface IR images of ATSC on a hot plate for 10 minutes in dry b) and wet c) states, demonstrating ATSC has good heat localization capability. White dashed lines indicate ATSC and hot plate. The thermal management performance of the ATSC under one sun radiation, taking a 2D root evaporator as control: d) The IR image of the ATSC under simulated sunlight in pure water, showing the temperature was not uniformly distributed across the entire structure and the temperature of the leaf portion was higher than the trunk portion. e) The temperature evolution profiles of different positions in ATSC from 0 to 3600 s, show an interfacial heating model. f) The IR image of a 2D root evaporator under one sun radiation in pure water. g) Temperature change profiles of the 2D root evaporator (top surface) and the underlying bulk water in 1 h. The insets in e) and g) are optical images of the actual device tested which uses thermocouples to measure temperature.

High‐efficiency light absorption is the primary requirement for maximizing evaporation performance for solar brine crystallizers. Therefore, the sunlight absorption property of the ATSC was evaluated. **Figure** **3**a shows the ATSC absorbed ≈94% of the incident light over the entire wavelength range of the solar spectrum due to the synergistic effects of the inherent black property of the CB and the light‐trapping property of the cellular structure.

In addition to water transport and sunlight absorption, the thermal localization and photothermal conversion capabilities of the crystallizer are also critical. Therefore, we tested the temperature increase of the ATSC in a dry state on a hot plate (60 °C) for 10 min (Figure 3b), and we found that the average temperature of the trunk only increased from 26.2 to 30.8 °C. Figure 3c shows that the surface temperature of the wetted ATSC, especially the leaf and trunk portions, almost no increase after heating for 10 min and even lower than environmental temperature, indicating the good heat‐trapping and thermal management ability of ATSC. The above results benefit from the low thermal conductivity of CB‐coated ATSC, thus ensuring that a large amount of heat is localized at the evaporation interface rather than dissipating to the underlying bulk water, thereby enhancing the evaporation performance.

Here we used the 3D ATSC and a 2D counterpart evaporator as two different solar crystallizers to measure their temperature distribution in pure water under one sun radiation by thermocouples. In the case of the ATSC, the temperature was not uniformly distributed across the entire structure and the temperature of the leaf portion was higher than the trunk portion (Figure 3d). As illustrated in Figure 3e, the temperature of the leaf portion (position b) was increased from ≈18 to ≈31 °C after 1 h of operation due to the continuous heat input. However, the bulk water (position e) temperature only increased from ≈19 to ≈21 °C, thus confirming the suppression of heat dissipation into the bulk water because of little contact area with water. Temperature evolution of the leaf portion (position a) shows the fast response of the absorber (steady‐state value of 24 °C in 7 min), which is attributed to the good photothermal conversion capability. Besides, the huge temperature difference between the evaporation layer and the bulk water shows an interfacial heating model. Although the temperature of the ATSC's side surface rose with increased radiation time, the temperature of the majority of the side surface remains lower than the surrounding environment temperature (25 °C) due to water transportation and evaporation from the side surface. Additionally, the temperature of ATSC in pure water under dark conditions was also much lower than the ambient temperature (Figure S5, Supporting Information). Under the above conditions, the temperature of the side surface of ATSC was lower than the environmental temperature due to evaporative cooling, thus it is possible for ATSC to absorb heat from the air via heat convection, conduction, and radiation, further enhancing evaporation performance.^[^ ^51^ ^]^

Figure 3f shows the temperature distribution of the 2D root evaporator in pure water under one sun radiation. The corresponding temperature variation from 0 to 60 min, including the top photothermal surface and the bottom bulk water, were presented in Figure 3g. The top surface of the 2D root evaporator was heated up to ≈33 °C within 7 min and finally reached a steady–state temperature of 34 °C. It was over 11 °C higher than that of bulk water, suggesting it can localize heat at the air/water evaporative interface. Compared with the 2D root evaporator, the 3D tree structure used by ATSC has a greater actual evaporation area and the temperature of its side surface is lower than ambient temperature, thus it may absorb extra energy from the environment. In conclusion, this ATSC has excellent solar absorption efficiency, good heat localization, and outstanding photothermal conversion capability.

### 2.4 Solar‐Driven Water Evaporation Performance of ATSC in the Different Source Water

**Figure 4:**
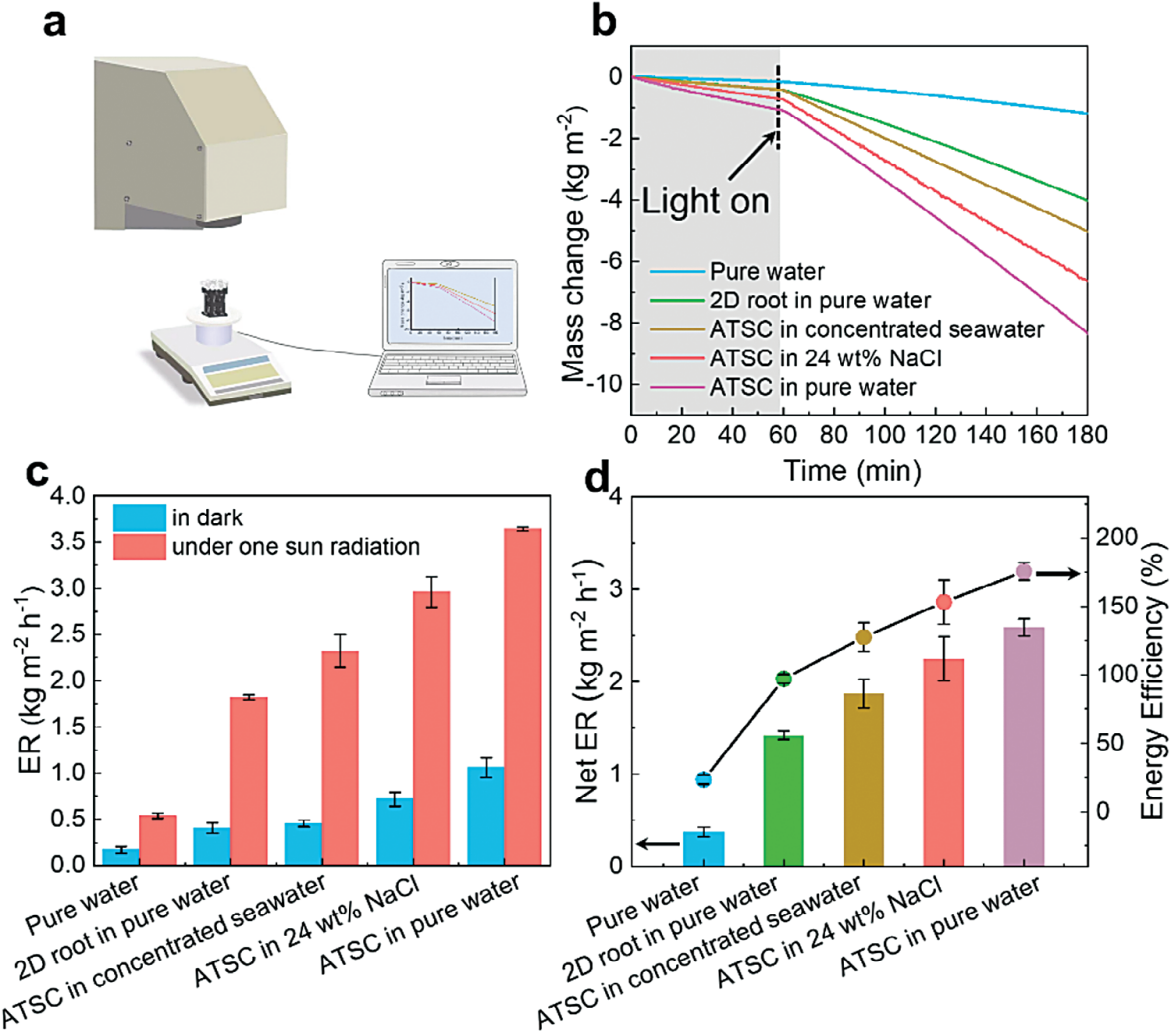
The water evaporation performance of ATSC and 2D root crystallizer in various source water. a) The scheme of the lab‐made setup for solar evaporation performance measurement, including solar simulator, solar crystallizer, electrical balance, and computer. b) The vapor generation performance in the different source water was calculated by monitoring the mass loss of source water for 3 h, with pure water as control, in which 1 h of the dark experiment and 2 h of the light experiment. c) The corresponding water evaporation rate (ER) under dark conditions and one sun radiation of ATSC and 2D root crystallizer in the different source water. The error bars in the ER resulted from environmental disturbance. Each error bar represents the standard deviation of at least five data points. d) The net ER, which subtracts the water ER in darkness from the solar steam generation rate under one sun radiation, and corresponding energy efficiencies. The error bars in the energy efficiency values resulted from errors in the measurement of the interface temperature, solar illumination power and the ER. Each error bar represents the standard deviation of at least five data points.

A lab‐made setup with one sun radiation was used to assess the performance of solar‐driven water evaporation (**Figure** **4**a). We prepared three ATSC with a distinct D of 0.45, 0.50, and 0.55 mm, respectively. Figure S6 (Supporting Information) reveals that the ATSC constituted of 0.55 mm struct has the highest evaporation rate (ER) in pure water, thus we use 0.55 mm as D to construct the 3D tree‐inspired ATSC and 2D root evaporator and compare their evaporation performance in the various source water (Figure 4b). The ER of our evaporators is described by the equation $\dot{m}=\frac{dm}{A\times dt}\;$, where *m* is the mass of evaporated water, *t* is time and *A* is the projected ground area.^[^ ^52^ ^]^ Furthermore, we designed and fabricated a heterogeneous artificial tree solar crystallizer (H‐ATSC) with different D. The root, trunk and leaf portions were composed of unit cells with D of 0.45, 0.50 and 0.55 mm, respectively (Figure S7a,b, Supporting Information). The water ER of H‐ATSC was found to be similar to that of the homogeneous cellular structure (viz. ATSC) (Figure S7c,d, Supporting Information) since they have similar performance in terms of water transportation, photothermal conversion and thermal management. Consequently, we used the homogeneous cellular structure (viz. ATSC) owing to its structural simplicity as the solar crystallizer for the subsequent wastewater treatment experiments. As Figure 4c shows, in the darkness, the ER of pure water is 0.15 kg m^−2^ h^−1^, and it became more pronounced with the incorporation of evaporators to 0.42 kg m^−2^ h^−1^ for the 2D root crystallizer and 1.07 kg m^−2^ h^−1^ for ATSC, due to the highly increased total surface area available for evaporation by the trunk and leaf portions of the ATSC for at least 3.4 times larger than the projected area of 2D root crystallizer. Similarly, under one sun radiation, the steam generation rate increases from 0.51 kg m^−2^ h^−1^ for pure water to 1.82 kg m^−2^ h^−1^ for the 2D root crystallizer in pure water and 3.64 kg m^−2^ h^−1^ for ATSC under the same conditions. Currently, many interfacial solar crystallizers use NaCl solution to simulate seawater, while treating real seawater, the capacity to evaporate water was almost lost due to the crystallization of high‐valent cations hindering the water‐wicking channels. To investigate the feasibility of ATSC in actual high‐salinity wastewater, 24 wt.% NaCl aqueous solution and concentrated seawater from natural seawater after concentration were chosen for evaporation experiments. Besides sodium and chlorine, the other primary ions in real seawater include magnesium, calcium, and potassium. The concentration of Na^+^ increased from 8.8 g L^−1^ in natural seawater to 54.0 g L^−1^ after concentration. Meantime, the concentrations of Mg^2+^, Ca^2+^, and K^+^ were 5.3, 1.3, and 4.0 g L^−1^, respectively in concentrated seawater (Table S1, Supporting Information). The ER of ATSC under one sun radiation in the 24 wt.% NaCl solution (2.96 kg m^−2^ h^−1^) and the concentrated seawater (2.30 kg m^−2^ h^−1^) is lower than in pure water (3.64 kg m^−2^ h^−1^), which can be ascribed to the lower water vapor pressure at the evaporative interface of the saline water, thus lowering the driving force for evaporation and decreasing the solar steam generation rate.

The net ER is the difference between the solar steam generation rate under one sun radiation and the water evaporation rate in darkness. The energy efficiency of the solar crystallizer was calculated as the percentage of the energy that is utilized by net ER compared with the total energy of the incident sunlight to evaluate the photothermal conversion performance (calculation details in Supporting Information Section 2.1). The ATSC presents ultra‐high energy efficiency when treating various source water (128% of concentrated seawater, 150% of 24 wt.% NaCl, 175% of pure water) compared with the 2D root crystallizer (96% of pure water) (Figure 4d), which can be attributed to the environmental energy‐enhanced effect and the vast effective evaporation area. The corresponding heat loss analysis of the evaporation process of ATSC in pure water under radiation can be found in the Supporting Information (Section 2.2). To investigate the evaporation contribution of the leaf and trunk portions to the overall ATSC, we used some plastic wrap to cover the trunk portion of ATSC, which is denoted as ATSC‐NT, to avoid vapor escape from trunk portion and then we measured its evaporation performance (Figure S8a,b, Supporting Information). The weight change of the ATSC‐NT and ATSC in pure water was recorded in real‐time (Figure S8c, Supporting Information). After analyzing, we found the ER of ATSC‐NT was lower than that of ATSC due to the reduced evaporation area of ATSC‐NT. Specifically, the solar steam generation rate under one solar radiation for the ATSC‐NT and ATSC was 2.66 and 3.64 kg m^−2^ h^−1^, respectively (Figure S8d, Supporting Information), which means the trunk contributed 26.9% ER of the whole ATSC. In conclusion, the ATSC with advanced designs in structures and materials can enable effective suppression of heat loss through conduction and energy gain from the environment. Hence, the overall evaporation performance was significantly improved.

### 2.5 Solar Crystallization of ATSC in Real Seawater Brine

**Figure 5:**
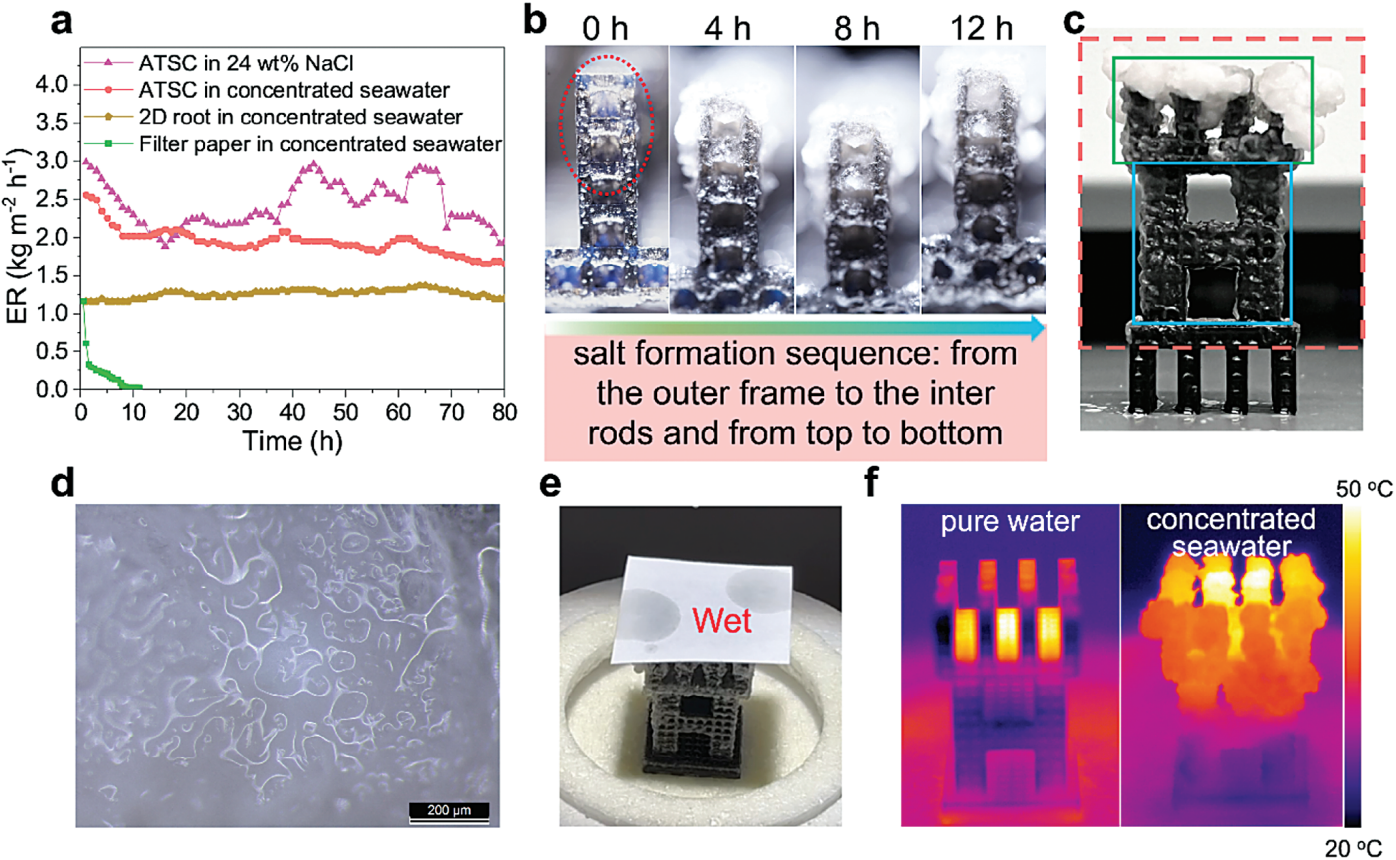
Long‐term evaporation performance and crystallization behavior of ATSC in high‐salinity brine. a) Water evaporation endurance test of different solar crystallizers in various high‐salinity brine under one sun radiation for 80 h (each point on the curve was the average ER of 1 h). b) The salt formation process of the ATSC under light radiation in concentrated seawater for 12 h, indicating the salt crystallization was from the outer frame to the inter rods and from top to bottom. c) The corresponding digital photo of the ATSC after 80 h of exposure using concentrated seawater. The red dashed lines mark the evaporation area, and the green and blue boxes are the leaf and trunk portions of the ATSC, respectively. d) An optical microscope image of salt accumulated on ATSC after operating with concentrated seawater for 80 h, indicating the salt is rough and highly porous. e) When a piece of filter paper was brought into contact with the salt layer, water can be absorbed from the salt, indicating the salt layer deposited on the ATSC is wet, thus the salt crust layer does not affect the water transport ability. f) The temperature distribution of ATSC in pure water (left) and in concentrated seawater (right) after radiation for 80 h, indicates partial areas in the leaf portion are still available for photothermal conversion.

To further investigate the long‐term operational stability, the different solar crystallizers in various high‐salinity brine were continuously radiated by the simulated sunlight for 80 h in lab conditions. There was no manual salt removal from the solar crystallizer during the whole evaporation process. For comparison, a typical interfacial evaporation system was built with CB‐coated commercial filter paper (diameter of 90 mm) as a photothermal layer and a hydrophilic cotton rod to deliver water (Figure S9, Supporting Information). **Figure** **5**a shows the ER of the conventional solar crystallizer quickly decreased by 48% in 0.5 h and plummeted to near zero water evaporation by the end of 9 h. The reason for the rapid decline of the evaporation performance of the conventional solar crystallizer is that the densely packed salt formed in the cotton rod during evaporation hinders water transportation for continuous evaporation (Figure S10, Supporting Information). During the whole testing period, the performance of the 2D root crystallizer in concentrated seawater, ATSC in concentrated seawater and 24 wt.% NaCl brine was largely stable, and the average ER of 1.26, 1.94, and 2.42 kg m^−2^ h^−1^ were achieved, respectively. As mentioned above, the effective evaporation area of the 2D root crystallizer is smaller than those of the ATSC, resulting in lower ER. Furthermore, the vapor pressure of concentrated seawater is lower than that of 24 wt.% NaCl brine, thus decreasing the ER. During 80 h of operation in high‐salinity brine, the ATSC and 2D root crystallizer were gradually covered with a salt layer (Figure S11, Supporting Information). Since the brine is not saturated, including 24 wt.% NaCl and concentrated seawater, the salt crystal formed in the early stage may re‐dissolve or collapse when the water wicks to that area again (Figure S12, Supporting Information). This phenomenon coupled with environmental disturbances can explain some fluctuation in the ER during the long‐time operation. Compared to treating the concentrated seawater, the salt deposited on the ATSC was faster when treating 24 wt.% NaCl brine, resulting in a more drastic ER curve.

It is interesting to note that with much salt accumulation on these solar crystallizers during 80 h of operation, the ER was almost constant^[^ ^53^ ^]^ and retained a high ER over 1.0 kg m^−2^ h^−1^ (except the filter paper) in various high‐salinity brine, thus maintaining a durable water evaporation performance. Compared with the reported 3D evaporators and solar crystallizers, the comprehensive performance (salinity, evaporation rate and duration) of ATSC is optimal (Table S2, Supporting Information). For each BCC (with added frame) unit cell, since the evaporation of water only occurs on the outer surface composed of the outer framework, the salt concentration on the outer surface gradually concentrates until it reaches saturation and eventually crystallizes. Therefore, the salt preferentially accumulated on the outer frame of the unit cell instead of the inter rods thus achieving the separation of the crystallization interface and the water transportation pathways. Such a phenomenon is proven by Figure 5b and Figure S13 (Supporting Information). Inside one unit cell, salt crystals first nucleate on the structs, and then with continuous exposure to the light, the salt crystals gradually grow to block the micrometer‐sized cavities formed between the crossed structs (Figure S14a, Supporting Information), and finally, the salt blocks the millimeter‐sized cavities on the end face of the unit cell (Figure S14b, Supporting Information). Additionally, for the overall tree structure of ATSC, as the leaf portion is closer to the light source and therefore has a higher surface temperature than the trunk portion (Figure 3e), the salt preferentially crystallizes on the leaf portion, while the trunk portion has very little salt crystallization (Figure 5c), finally ensuring sufficient water supply for the whole tree structure. Specifically, there was a small amount of salt formation on the trunk portion of ATSC after 24 h while salt accumulation on the leaf portion was substantial. 72 h later, a large amount of salt particles formed on the leaf portion due to the crystallization of salt, while a thin salt crust layer formed on the trunk portion of ATSC, covering its external surface (Figure S14c, Supporting Information). Meanwhile, the trunk portion also provides a certain evaporation surface, which contributes 26.9% ER of the whole ATSC (Figure S8, Supporting Information). During the longtime radiation, the ER of ATSC in concentrated seawater decreased from 2.55 to 1.65 kg m^−2^ h^−1^. In contrast to salt‐resistant evaporators, although the water evaporation performance of solar crystallizers can degrade after prolonged salt accumulation, it has the advantage of simultaneously harvesting clean water and solid salts, resulting in ZLD that eliminates liquid waste and maximizes water usage efficiency. Compared to ATSC without salt formation (2.55 kg m^−2^ h^−1^), the ER with salt accumulation after stabilization (1.65 kg m^−2^ h^−1^) only decreased by 35%, indicating the leaf portion with much salt accumulated was still evaporating. The above results prove that the strategy based on separating the crystallization interface and water‐wicking channel as well as the salt formation sequence from the outer frame to the inter rods and from top to bottom are the main reasons for the stable and ultra‐efficient crystallization of ATSC in concentrated real seawater.

The structure of the salt crystal collected from the ATSC when treating concentrated seawater was investigated using SEM observation with energy‐dispersive X‐ray spectroscopy (EDS) elemental mapping analysis. Figure S15 (Supporting Information) shows that the salt crust layer contained sodium, potassium, chlorine, magnesium, calcium, sulfur, and oxygen elements. Figure S16 (Supporting Information) shows that the salt crystals were piled up in a mixed mode and some micron‐scale pores were still present, which indicates that the formed salt crust layer was not very dense. Then we used optical microscopy to observe the morphology of salts on different parts of the ATSC. Figure S17 (Supporting Information) shows the salt accumulated on the leaf portion is thicker and whiter due to more salt accumulated than on the trunk portion. Moreover, the formed salt crust layer no matter on the leaf or trunk portion of ATSC exhibits a rough surface and loose porosity. The Kelvin equation^[^ ^54^ ^]^ predicted that evaporation would occur more quickly on the convex/planar salt crystals formed over the ASTC surface than on the concave water surface formed within the pores,^[^ ^55^ ^]^ leading to open pore formation in the crystallization front as salt crystals continued to grow on the convex salt extrudes. Similar preferential sites for salt accumulation are also observed in the process of water evaporation from porous media.^[^ ^56^, ^57^ ^]^ Figure S18 (Supporting Information) shows the salt crystals formed after various durations of light exposure. As can be seen, all salt crystals have rough surfaces and multiple layers of interconnected pores. Water can transport through these pores in salt crystals, giving the salt crystals their transparent color. As Figure S19a,b (Supporting Information) show, magnesium sulfate tended to grow in the voids between sodium chloride crystals, and the surface morphology of the two crystals was very different. The former had a rough, uneven, multilayered surface with multiple cracks on the scale of a few hundred nanometers to a few micrometers (Figure S19c, Supporting Information), whereas the latter had a smooth and flat surface. Figure S19d (Supporting Information) further indicates that magnesium sulfate and sodium chloride crystals mixed in a porous structure. It is believed that nano‐/micro‐pores of salt crystals were formed by the following mechanisms: 1) as Kelvin equation predicts, salt crystal nucleation and growth on previously precipitated crystal surfaces, leading to open pore formation in the crystallization front; 2) the presence of rough and often cracking magnesium sulfate at the interstitial spaces between the sodium chloride crystals promoted the formation of nano‐/micro‐pores. In contrast to most solar crystallizers that allow salt to grow on flat surfaces, our ATSC had open pores along with the cellular structure, which enabled the salt crystals to develop in 3D space^[^ ^58^, ^59^ ^]^ and have 10–100 µm pores for the transport of water (Figure 5d). This also provides additional evaporation area, thus compensating for the reduced ER due to salt coverage of the original evaporation surface, and maintaining a stable ER for a long time even with a large amount of salt accumulation. In summary, when treating concentrated seawater, the salt crystallized on the ATSC was highly porous, independent of the location of the salt crystallization (inside the unit cell or along the external cellular structure, leaf portion or trunk portion) and the duration of light exposure.

After 80 h of radiation in concentrated seawater, we used filter paper to contact ATSC. Figure 5e demonstrates that the salt layer that was produced on the upper surface of the leaf portion was wet. Moreover, the salt on the trunk portion was also wet (Figure S20, Supporting Information), indicating the salt layer does not affect the water transport capability of ATSC. Furthermore, it has been documented that when the water delivery channel in the crystallizer is nanoscale, Mg^2+^ and Ca^2+^ will block the channel during the evaporation of high‐salinity brine.^[^ ^60^ ^]^ In contrast, the water channels in ATSC are all in the micron size and the salt layer contains 10–100 µm pores, so the water transportation capability is hardly affected by ion plugging and salt crystallization. Thermal images show that the surface temperature of the ATSC during the solar evaporation experiment using concentrated seawater (right of Figure 5f, 36–50 °C) was higher than that using pure water (left of Figure 5f, < 35 °C), indicating that a large portion of light was still able to transmit through the salt crystals accumulated on the ATSC for absorption by CB. The solar absorption spectra of the salt crystals collected from ATSC show pure salt crystals possess a high reflectance (> 80%) in the short wavelength range (< 1300 nm) and a slightly lower reflectance (40%–90%) in the long wavelength range, which demonstrates poor solar absorption and energy utilization due to its inherent white color and the poor light‐trapping property (Figure S21, Supporting Information). This further suggests that the salt crystals on the surface of the ATSC are almost incapable of absorbing light, while light can reach the surface of the open‐cell cellular structure through the diffuse reflection and scattering and finally be converted into heat, leading to a minimal effect of the accumulated salt on the light absorption efficiency of the ATSC. Moreover, we also studied the solar absorption spectra of the ATSC with salts. Figure S22 (Supporting Information) indicates that the ATSC with salt has a similar light absorption efficiency with ATSC, especially in the UV–vis region. In other words, the formation of a salt crust layer did not significantly decrease the light absorption efficiency of the ATSC. These results demonstrate that the ATSC has the potential for continuously stable crystallization toward high‐salinity real brine for the ZLD goal.

### 2.6 Feasibility Under Practical Conditions

**Figure 6:**
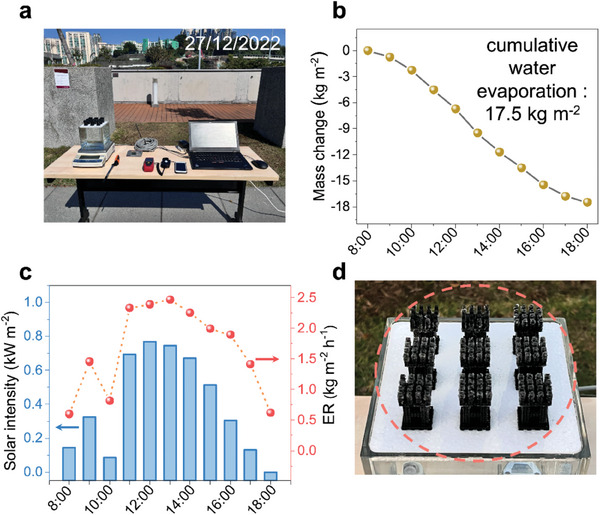
Outdoor experiments with the arrayed ATSC under natural sunlight. a) Digital image of the outdoor evaporation experiments module setup, including arrayed ATSC, electronic balance, temperature and relative humidity sensors, solar power meter, wind speed sensor, and a laptop computer. b) The cumulative mass change of concentrated seawater during 8:00–18:00. c) The solar intensity of outdoor environments during 10 h of operation and the corresponding ER of the arrayed ATSC. d) Photograph of the arrayed ATSC after the one‐day operation, many salts formed over the arrayed ATSC, indicating this device has good salt crystallization performance.

To verify the solar crystallization capability of ATSC in practical applications, we performed outdoor evaporation experiments with an easy‐to‐implement scale‐up solution and use concentrated real seawater as the source brine. **Figure** **6**a shows nine ATSC were assembled to form an array (with inter‐device space of 21 mm uniformly), which was tested on the campus rooftop on December 27, 2022, from 8:00 to 18:00. Moreover, the real‐time temperature, relative humidity, and natural wind velocity variation of the field test were also recorded in Figure S23 (Supporting Information). The cumulative weight change of the concentrated seawater during the 10 h test period was 17.5 kg m^−2^ (Figure 6b), indicating its potential for high efficiency and sustainable brine treatment in the future.

Figure 6c shows that the natural solar intensity increased to the highest 0.77 kW m^−2^ at noon. It is worth noting that the sun ≈10:00 was blocked by nearby buildings, thus there was a paradoxical drop in solar intensity and therefore ER, which was consistent with the results for temperature and relative humidity in Figure S23 (Supporting Information). Additionally, the arrayed ATSC delivered an ultra‐high ER of 2.47 kg m^−2^ h^−1^ in concentrated seawater. Under field conditions, the solar incident angle, environment temperature, relative humidity, and natural wind all affect the evaporation performance of the solar crystallizer. The continuous air convection across the arrayed solar crystallizer improved ER, which explained why the ER of ATSC in outdoor experiments was higher than that in a laboratory under the same solar intensity, indicating the versatility of the environmental‐enhanced solar crystallizer. After 10 h of outdoor testing, all nine ATSC had apparent salt accumulation, especially on the leaf portion (Figure 6d), demonstrating that the array of ATSC has great potential for large‐scale salt production under natural sunlight.

## 3 Conclusion

Herein, we presented a rationally designed ATSC based on the cellular architecture composed of multiple unit cells of BCC (with added frame). CB‐coated ATSC demonstrated excellent evaporation performance owing to its vast surface area, fast water transport ability, enhanced light absorption efficiency and outstanding thermal management property, which make it possible to achieve long‐term stable ZLD treatment of real seawater brines. With the novel design of ATSC, the salt was preferentially crystallized on the outer frame rather than in the inner voids, ensuring the water‐wicking channels remain open after prolonged salt crystallization. In addition, when salt covered the leaf portion of ATSC in large amounts, the trunk portion was less salt crystallized and continued to have enough surface area for evaporation. Besides, the accumulated salt was irregular and highly porous, making ATSC sustain a stable and ultra‐high evaporation rate of 1.94 kg m^−2^ h^−1^ on average over 80 h in the real brine from concentrated seawater under one sun radiation. The designed structure presented in this paper represents a significant advancement toward ZLD treatment of high‐salinity brine in many industry processes, such as salt recovery from waste brines and salt mineral extraction from salt lakes.

## 4 Experimental Section

### Design and Fabrication of the Solar Brine Crystallizer

The model of the crystallizer was designed using SOLIDWORKS 2019 software. The tree‐shaped solar crystallizer consisted of three parts: the root, the trunk, and the leaf. The detailed crystallizer design is presented in Figure 1b. The root and the leaf had the same structure. The height of the root, trunk, and leaf was designed as 10.30, 20.05, and 10.30 mm, respectively, thus the total height is 40.65 mm. All parts with fine features were printed using High Temp Resin by a Form 3 printer (Formlabs) with 25 µm layers and post‐cured for 120 min at 80 °C while exposed to 405 nm light (Form Cure, Formlabs). The 3D‐printed structure was immersed in CB nanoparticles (2 g) dissolved in ethanol (100 mL) and taken out to allow it to dry in an oven (80 °C). This process was repeated 3 times and the CB loading amount was ≈90 mg per crystallizer.

### Preparation of Concentrated Real Seawater

The seawater (collected from Victoria Harbor, Hong Kong in December 2022) was concentrated in a blast oven at 85 °C and then filtrated by 0.45 µm cellulose acetate membrane. The detailed water quality of real seawater samples was shown in Table S1 (Supporting Information). During the concentration process of natural seawater, there were some white precipitates formed, which were removed by filtration. The XRD pattern of the precipitates (Figure S24, Supporting Information) indicates that their main compositions were calcium sulfate hydrate (CaSO_4_·2H_2_O) and calcium sulfate (CaSO_4_), which have poor solubility in water.

### Solar Evaporation and Crystallization Experiments

A lab‐scale setup was built to evaluate the solar evaporation and crystallization performance of the solar crystallizer. A solar simulator (Newport Oriel Solar Simulator, 94021A and power supply 69 907) equipped with AM 1.5G filter was used to provide solar radiation with a constant intensity of 1000 W m^−2^. The intensity of the solar radiation was measured by a solar power meter (Solar‐100, AMPROBE). The beam size (5 cm × 5 cm) was slightly larger than the photothermal material and exactly perpendicular to the solar crystallizer. An electronic analytical balance (ME204E, Mettler Toledo) with an accuracy of 0.1 mg was used to record the mass change of brine in real‐time which was then used to calculate the water evaporation rate. The temperature distribution was monitored by an IR camera (A600‐series, FLIR). The real‐time temperature distributions were measured using thermocouples (K type, KAIPUSEN) and recorded by a data logger (AT4208). During the water evaporation test, the crystallizer was embedded in the polystyrene (PS) foam to isolate the natural evaporation of bulk water. The evaporation rates of the solar crystallizer when treating various source water were measured in the dark for 1 h and under one sun radiation for 2 h. When treating concentrated seawater and 24 wt.% NaCl solution, there was no manual salt removal from the solar crystallizer unless otherwise specified. All lab measurements were conducted at an ambient temperature of 22–25 °C with a relative humidity of 55 ± 5% as monitored using a temperature and humidity meter (AZ88162). The outdoor field test was conducted on the rooftop of a housing unit inside The Hong Kong Polytechnic University campus on December 27, 2022.

## Conflict of Interest

The authors declare no conflict of interest.

## Supporting information

Supporting Information

## Data Availability

The data that support the findings of this study are available in the supplementary material of this article.
