# Supplementary material for: 3D Cellular Solar Crystallizer for Stable and Ultra‐Efficient High‐Salinity Wastewater Treatment
Source: Adv Sci (Weinh). 2023 Dec 1;11(2):2305313. doi: 10.1002/advs.202305313 (PMC10787074; doi:10.1002/advs.202305313)
Supplement: Supplementary file 1 — Supporting Information [file ADVS-11-2305313-s001.pdf]

ADVANCED SCIENCE
Open Access

Supporting Information

for *Adv. Sci.*, DOI 10.1002/advs.202305313

3D Cellular Solar Crystallizer for Stable and Ultra-Efficient High-Salinity Wastewater Treatment

*Can Wang, Hanchao Zhang, Zhanxiao Kang and Jintu Fan**

Supporting Information

Three-Dimensional Cellular Solar Crystallizer for Stable and Ultra-Efficient High-Salinity Wastewater Treatment

*Can Wang, Hanchao Zhang, Zhanxiao Kang, and Jintu Fan**

Research Centre of Textiles for Future Fashion
School of Fashion and Textiles
The Hong Kong Polytechnic University
Kowloon, Hong Kong 999077, China
E-mail: jin-tu.fan@polyu.edu.hk

1. Experimental section

1.1. Materials

Carbon black (CB) was supplied by Cabot Corporation (EMPEROR 2000). Sodium chloride and ethanol were analytical reagents purchased from Sigma-Aldrich. Polystyrene (PS) foam (thickness of 5 mm) was purchased from a local market. Filter paper (Whatman 6, Qualitative) was used to prepare a conventional solar crystallizer. All chemicals were used as received without further purification.

1.2. Characterizations

Field-emission scanning electron microscopy (FESEM) images were captured on a Tescan MIRA. Optical microscope photos were obtained by Leica M165 C stereo microscope. UV-Vis-NIR spectra of the solar crystallizer were recorded using a UV-3600i Plus spectrophotometer equipped with an integrating sphere (Shimadzu). The absorption spectra of the samples were calculated based on the reflectance (R%) and transmittance (T%) results. The light absorbance (A%) of the crystallizer was calculated by the equation: $A\% = 1 - R\% - T\%$. The water contact angles were analyzed by the dynamic contact angle tester (SDC-350). The water quality of natural seawater was measured by an inductively coupled plasma optical emission spectrometer (ICP-OES, Agilent 700 Series). A digital camera (Sony α7 III) was used to image the 3D solar crystallizer and the salt crystallization processes during the evaporation of brine. The powder X-ray diffraction (XRD) patterns were recorded on a Japan Rigaku Ultima IV. The X-ray photoelectron spectroscopy (XPS) was acquired using a Thermo Scientific K-Alpha Nexsa spectrometer. All salt crystal samples were dried in an oven at 40 ^{o}C for 4 hours before characterization.

2. Calculation section

2.1 Solar-to-vapor energy efficiency estimation

The energy efficiency (η)[1] of the solar crystallizer was generally calculated via the following equation (1) to (4):

$$\eta = m \times h_{LV}/P_{solar} \quad (1)$$

$$h_{LV} = Q_s + Q_l \quad (2)$$

$$Q_l = -0.00006 \times {T_i}^3 + 0.0016 \times {T_i}^2 - 2.36 \times T_i + 2500.8 \quad (3)$$

$$Q_s = c \times (T_i - T_w) \quad (4)$$

where m (kg m^{-2} h^{-1}) is the net ER; h_{LV} (kJ kg^{-1}) is the total enthalpy required for the water evaporation, which is the summary of the sensible heat (Q_s) required for the temperature increase and the latent heat (Q_l) required for the phase change; Q_l (kJ kg^{-1}) changes under different temperatures and the average temperature of the water/air interface where the vaporization occurs is used as T_i (^{o}C) in this case; Q_s (kJ kg^{-1}) is dependent on the T_i and the source water temperature T_w (^{o}C); c is the specific heat of water which is 4.2 kJ kg^{-1} ^{o}C^{-1}; and P_{solar} is the simulated solar energy input, which is 1 kW m^{-2} in our experiments.

2.2 Energy analysis of ATSC in the evaporation process

(a) The radiation heat loss to the ambient could be calculated by the Stefan-Boltzmann equation[2-4]:

$$P_{rad} = \varepsilon\sigma({T_1}^4 - {T_2}^4) \quad (5)$$

where P_{rad} denotes the radiation heat flux, ε is the emissivity of the wetted surface, σ is the Stefan-Boltzmann constant (5.67×10^{-8} W m^{-2} K^{-4}), T_1 is the temperature of the wetted surface and T_2 is the ambient temperature (25.0 ^{o}C) in our experiment. It is assumed that the wetted absorber has a maximum emissivity of 1. Since the wetted surface had an uneven temperature distribution, the average temperature (28.2 ^{o}C) was adopted for T_1 in the calculation.[5] Therefore, we can estimate that the radiation heat loss of the absorber accounts for ~ 2.0% of the incident solar energy.

(b) The convection heat loss could be expressed by Newton's law of cooling[6, 7]:

$$P_{conv} = h(T_1 - T_2) \quad (6)$$

where P_{conv} denotes the convection heat flux, h is the convection heat transfer coefficient (5 W m^{-2} K^{-1}),[8] T_1 is the average temperature of the wetted surface, and T_2 is the ambient temperature. In our experiments, the convection loss accounts for ~ 1.6% of the incident solar energy.

(c) The downward conduction heat loss from the absorber to water can be estimated by[9, 10]:

$$P_{cond} = \frac{\kappa(T_1 - T_w)}{l} \quad (7)$$

where κ is the thermal conductivity (0.20 W m^{-1} K^{-1}), T_1 is the average temperature of the wetted surface (28.2 ^{o}C), T_w is the temperature of source water (21.2 ^{o}C), and *l* is the heat conduction path referring to the height of ATSC (41 mm) in our experiment. Therefore, P_{cond} has accounted for ~ 3.4% of the incident solar energy.

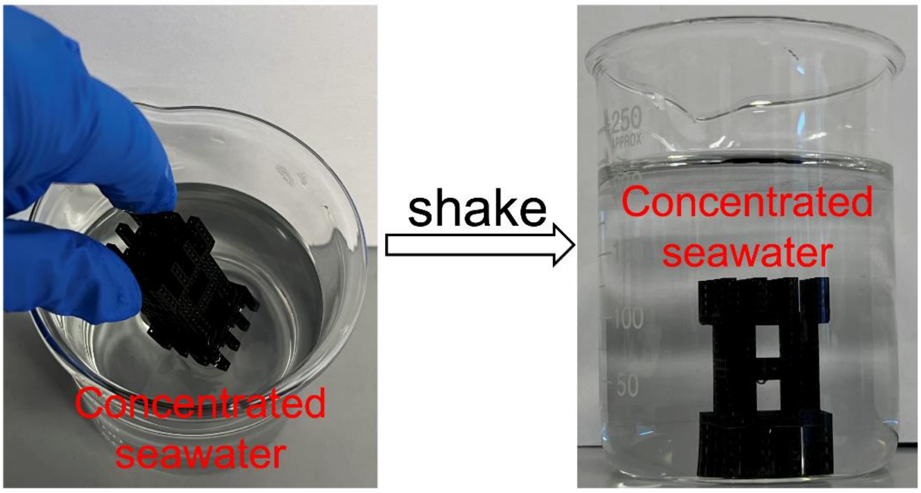

Figure S1. Stability of ATSC which was shaken for 5 minutes in concentrated seawater.

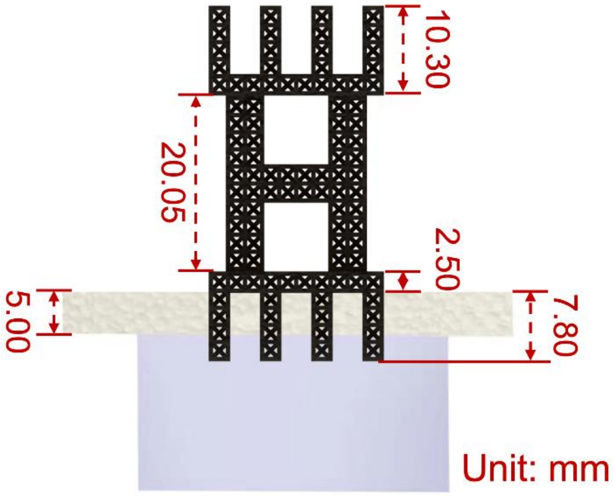

Figure S2. The configuration of the ATSC in PS foam.

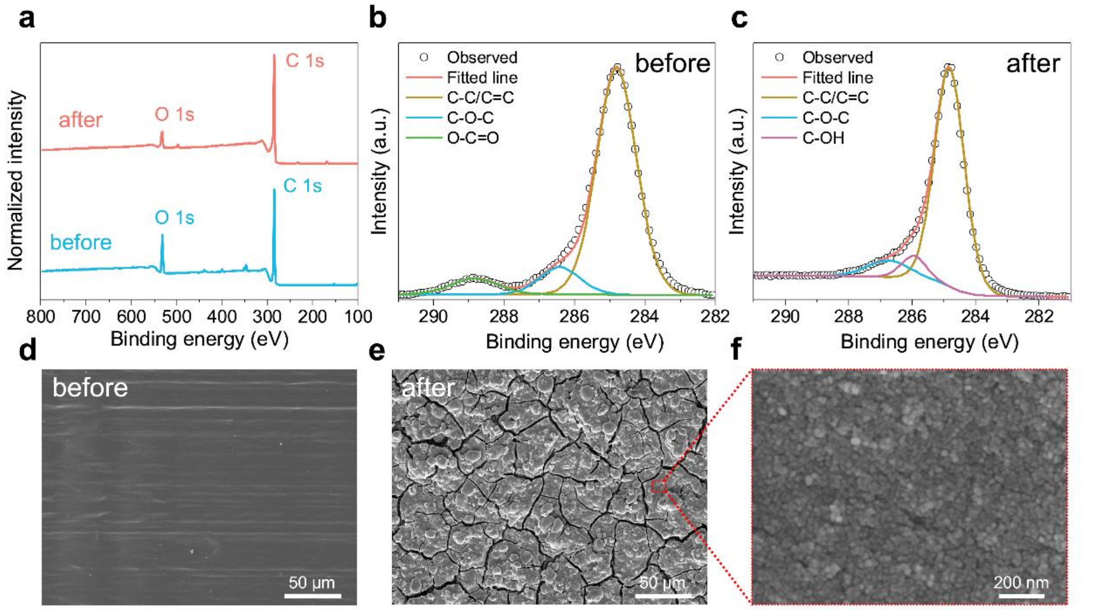

Figure S3. a) XPS patterns of ATSC before and after coating with CB. The atomic ratio of carbon increases from 83.04% to 88.71% after coating, which means that CB is successfully loaded to the ATSC. The C 1s XPS spectrum of ATSC before b) and after c) coating with CB. SEM images of ATSC before d) and after e) coating with CB. f) High-resolution SEM image of the CB nanoparticles.

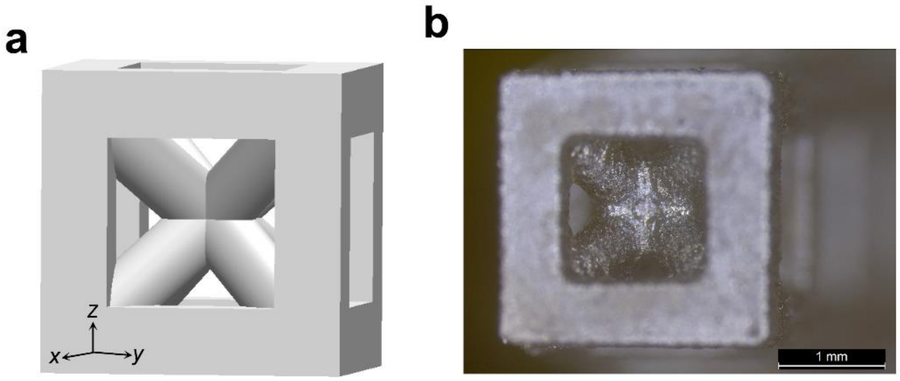

Figure S4. a) Diagram of one unit cell in ATSC. b) Microscopy image of one unit cell.

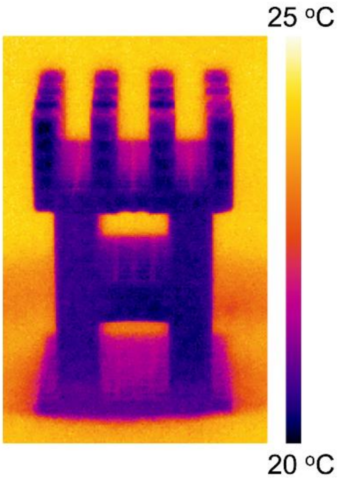

Figure S5. The IR image of ATSC in pure water under a dark condition.

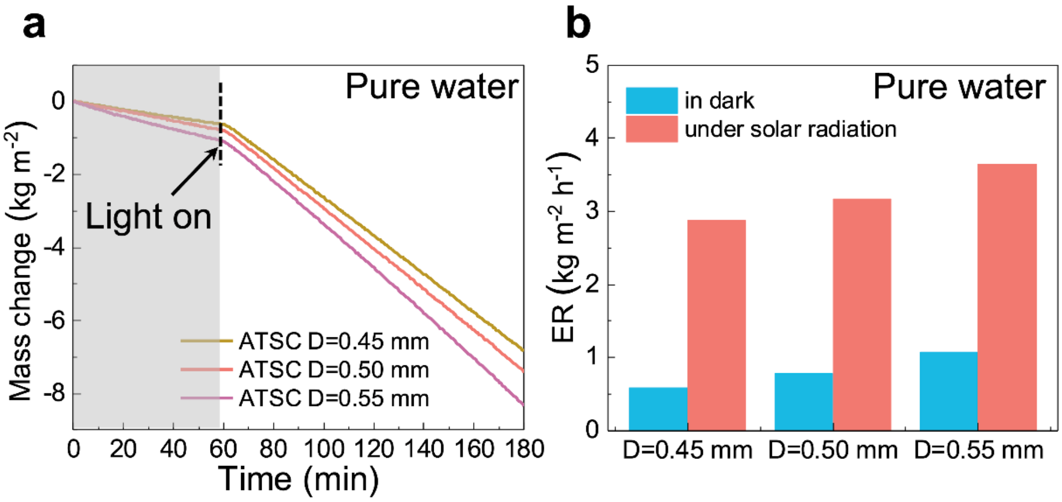

Figure S6. a) The mass change curves of ATSC in pure water, which is composed of different D. b) The corresponding ER of three ATSC with different D.

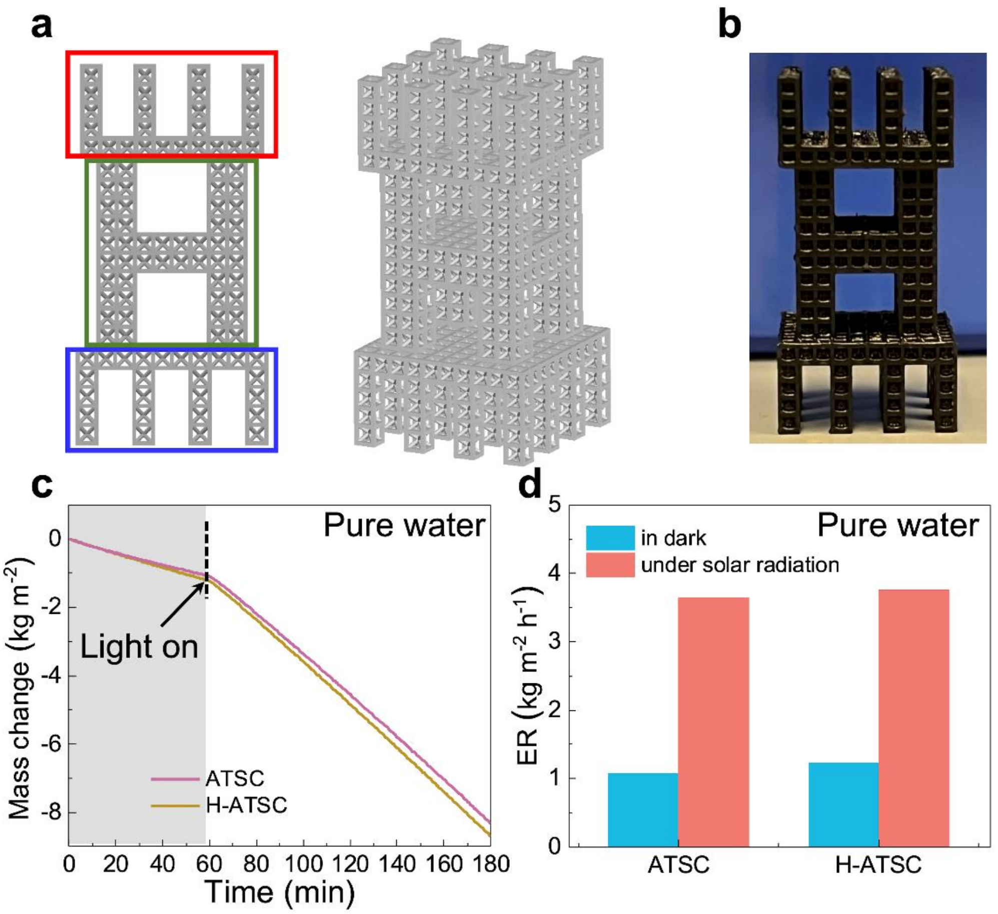

Figure S7. a) Schematic illustration of the H-ATSC. The root section, shown in the blue box, is made up of unit cells with a struct diameter D of 0.45 mm, the trunk portion, shown in the green box, is made up of unit cells with D of 0.50 mm, and the leaf portion, shown in the red box, is made up of unit cells with D of 0.55 mm. b) The optical diagram of the prepared H-ATSC. c) The mass change curves of ATSC and H-ATSC in pure water. d) The corresponding water ER of ATSC and H-ATSC in the dark and under solar radiation.

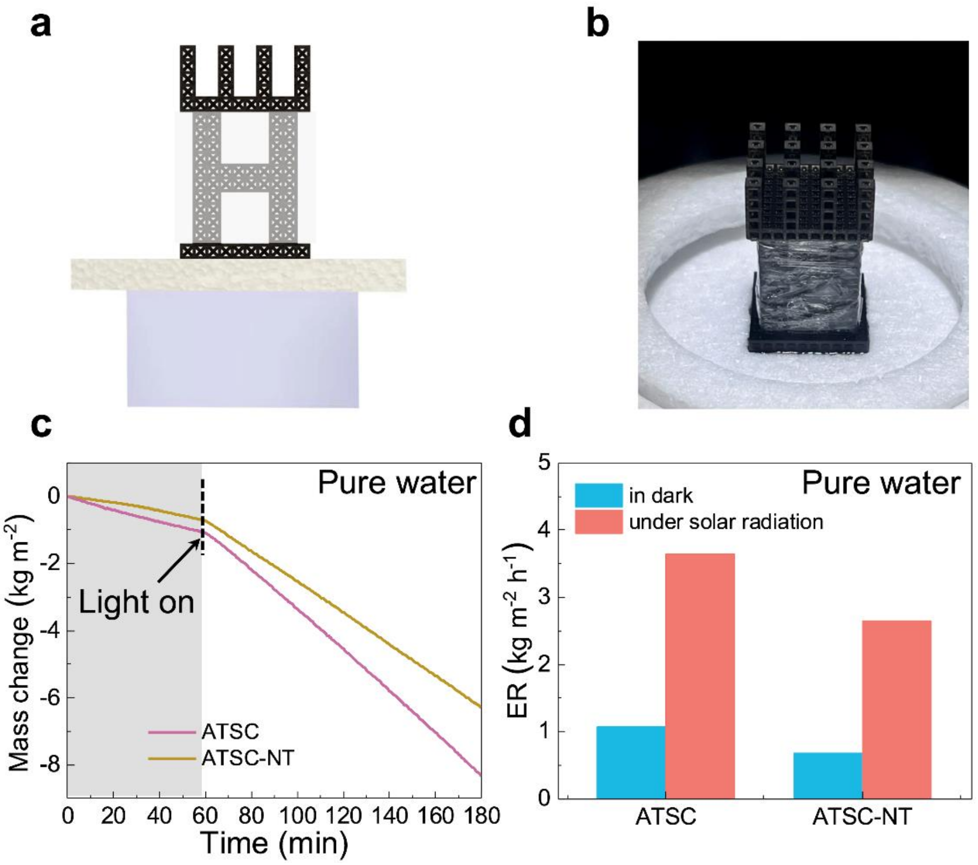

Figure S8. a) Schematic illustration of the ATSC-NT with plastic wrap to cover trunk portion which avoiding vapor escape from trunk portion. b) The optical diagram of the ATSC-NT for evaporation. c) The mass change curves of ATSC and ATSC-NT in pure water. (d) The corresponding water ER of ATSC and ATSC-NT in the dark and under solar radiation.

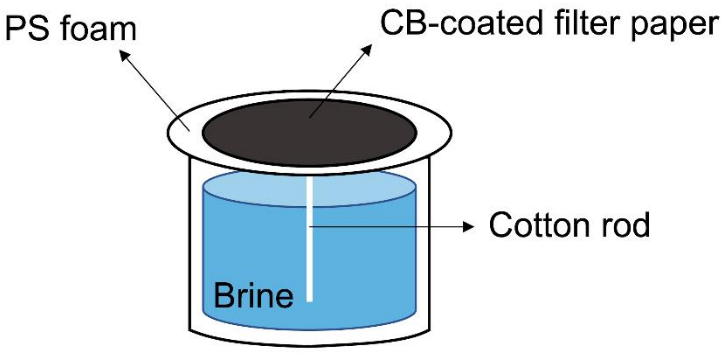

Figure S9. The schematic diagram of the conventional solar crystallizer. The CB-coated filter paper was placed on top of PS foam. Brine was transported from the water reservoir upward to CB-coated filter paper by using the hydrophilic cotton rod through a hole in the PS foam.

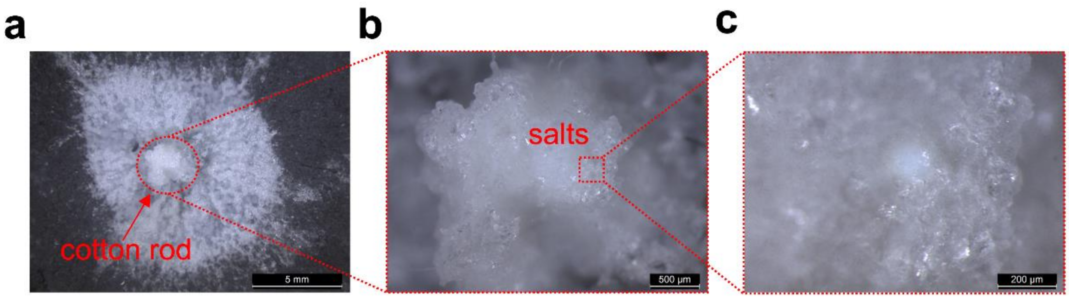

Figure S10. Optical microscope images of salt accumulated on filter paper after operating with concentrated seawater for 11 hours, showing the salt accumulated in the cotton rod hinders water transportation.

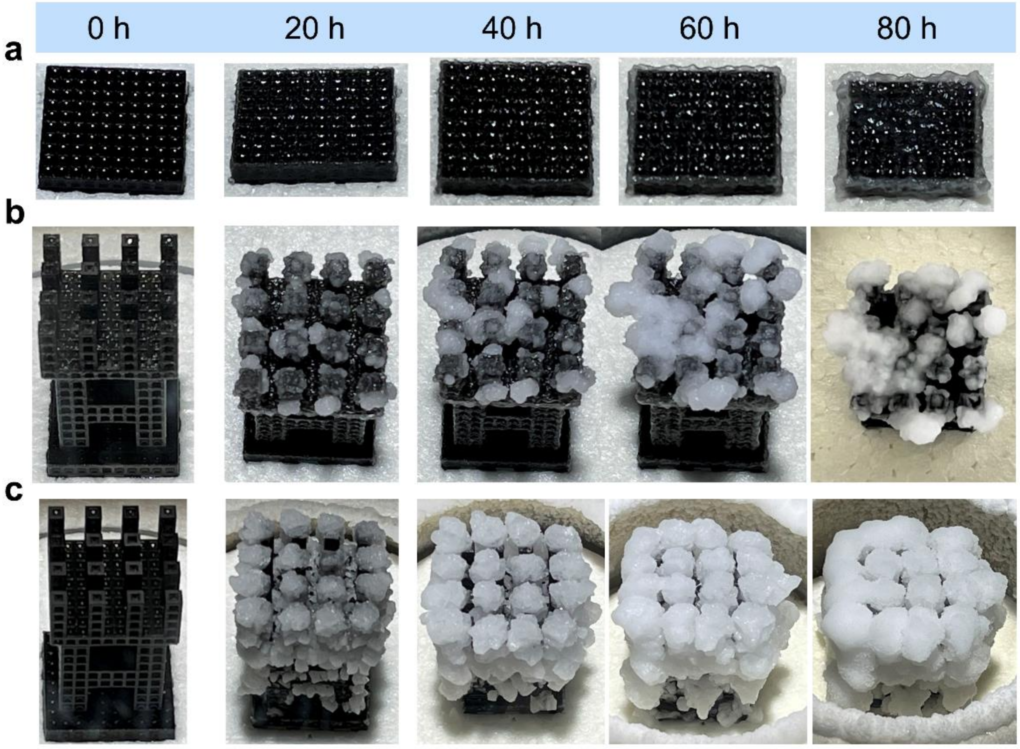

Figure S11. The optical photos of different solar crystallizers in high-salinity brine during radiation for 80 hours, including 2D root crystallizer in concentrated seawater a), ATSC in concentrated seawater b), and ATSC in 24 wt% NaCl solution c).

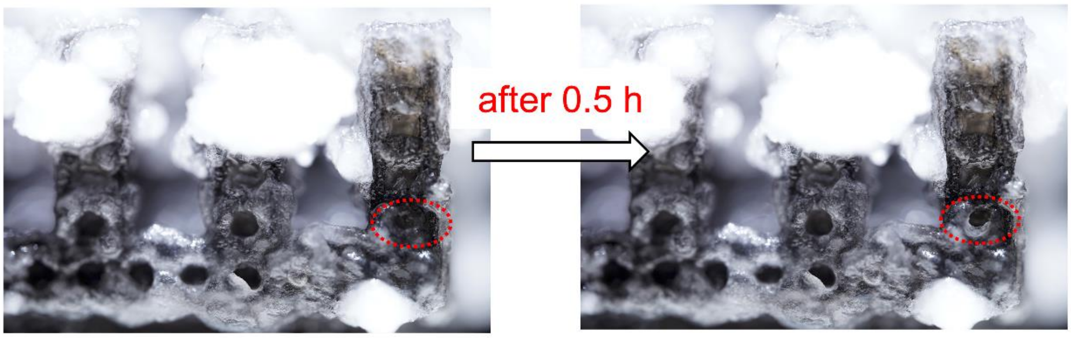

Figure S12. The photos of salts formed on ATSC when treating concentrated seawater indicate redissolution of the accumulated salt may occur.

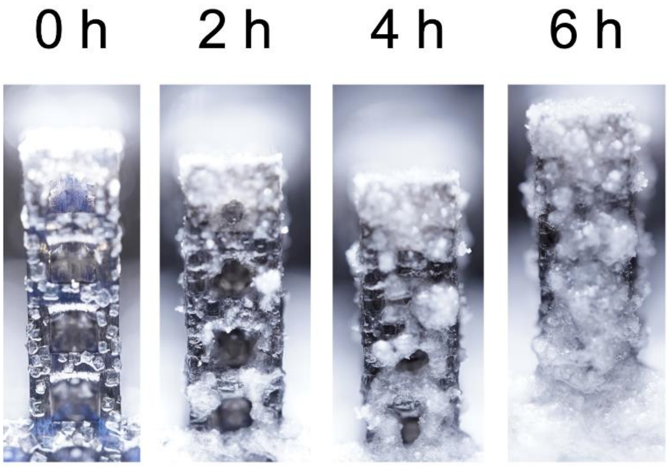

Figure S13. The salt formation process of the ATSC under continuous light radiation in 24 wt% NaCl solution, indicating the salt crystallization was from the outer frame to the inter rods and from top to bottom.

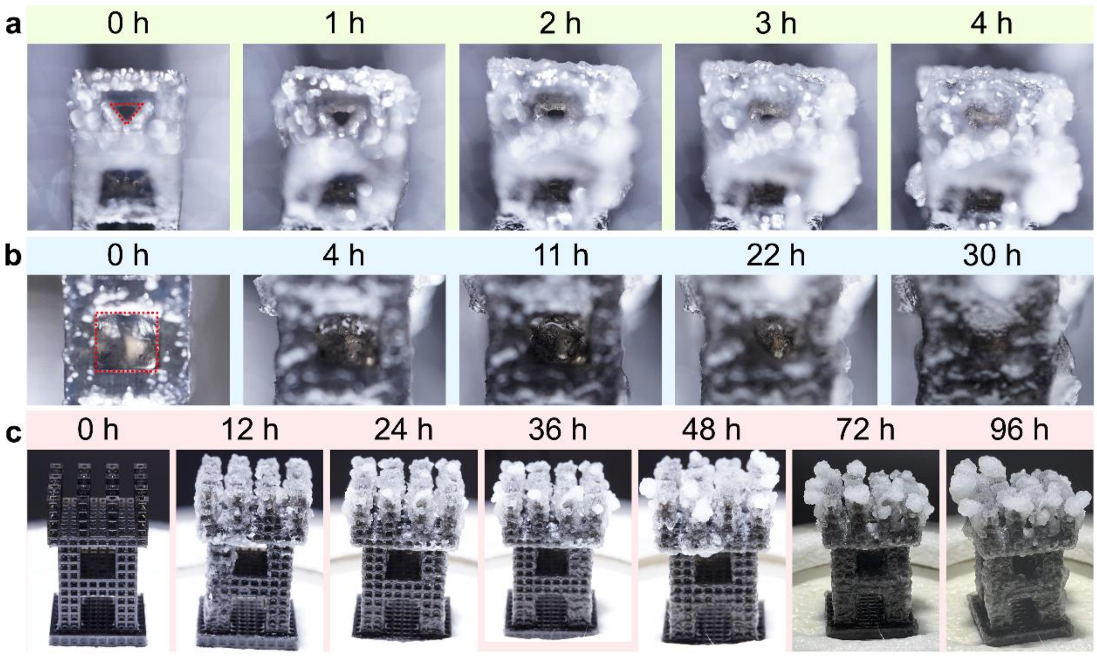

Figure S14. The optical photos of salt crystal nucleation and growth inside one CB-coated unit cell of ATSC when treating concentrated seawater under one solar radiation. a) The top view with the micrometer-sized cavity marked with a red triangle and b) the side view with the millimeter-sized cavity marked with a red square. c) The optical photos of the salt crystal growth process along the external surface of the cellular structure.

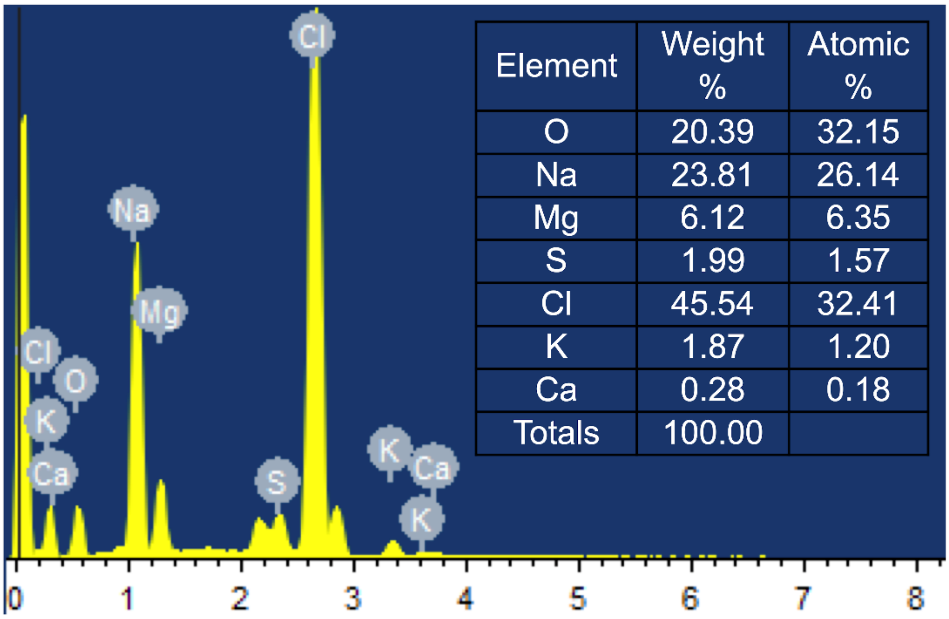

Element	Weight %	Atomic %
O	20.39	32.15
Na	23.81	26.14
Mg	6.12	6.35
S	1.99	1.57
Cl	45.54	32.41
K	1.87	1.20
Ca	0.28	0.18
Totals	100.00	

Figure S15. The energy-dispersive X-ray spectroscopy (EDS) analysis of the salt crust layer formed from the concentrated seawater. This salt crust layer contained oxygen, sodium, magnesium, sulfur, chlorine, potassium, and calcium elements.

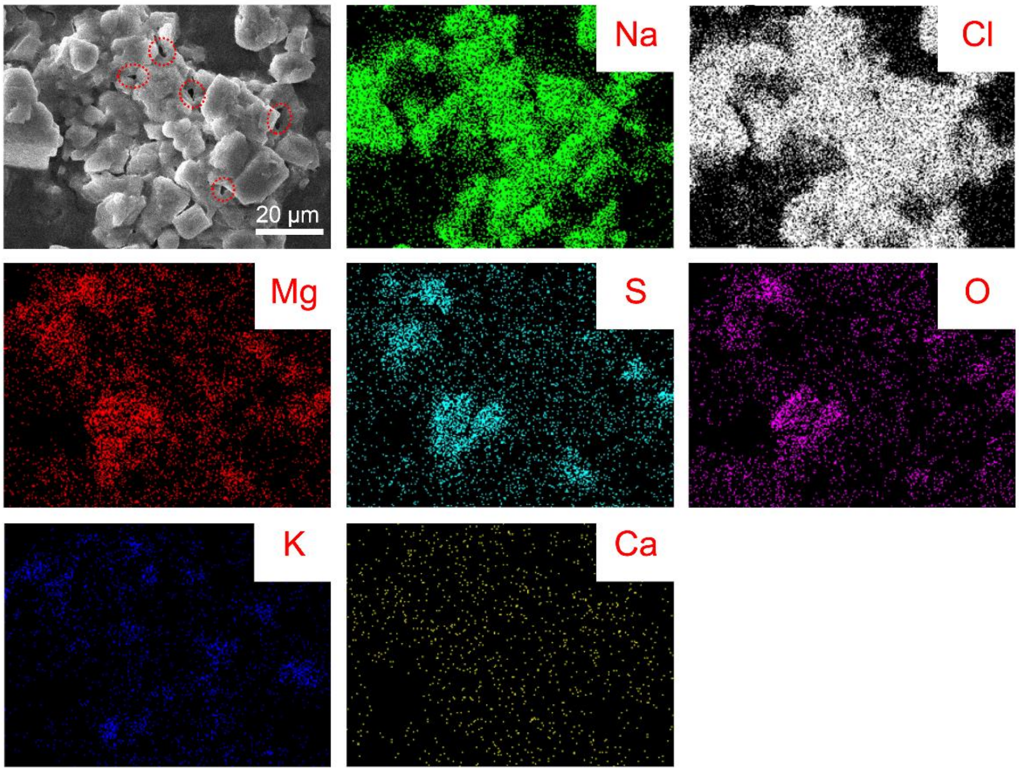

Figure S16. The morphology of the salt crystals collected from ATSC when treating concentrated seawater and the corresponding element distribution.

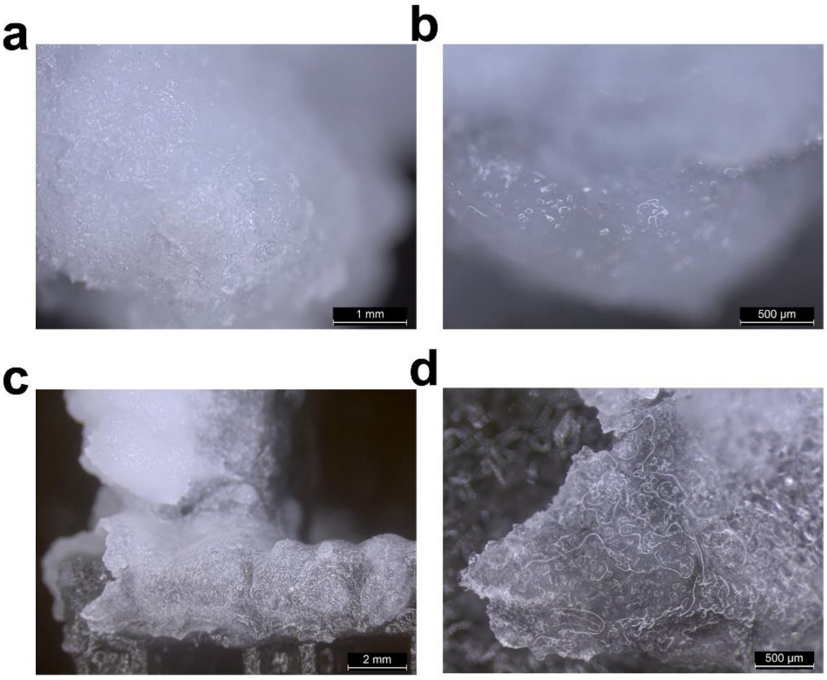

Figure S17. The morphology of salts collected from different portions of the ATSC: the leaf portion (a and b), the trunk portion (c and d).

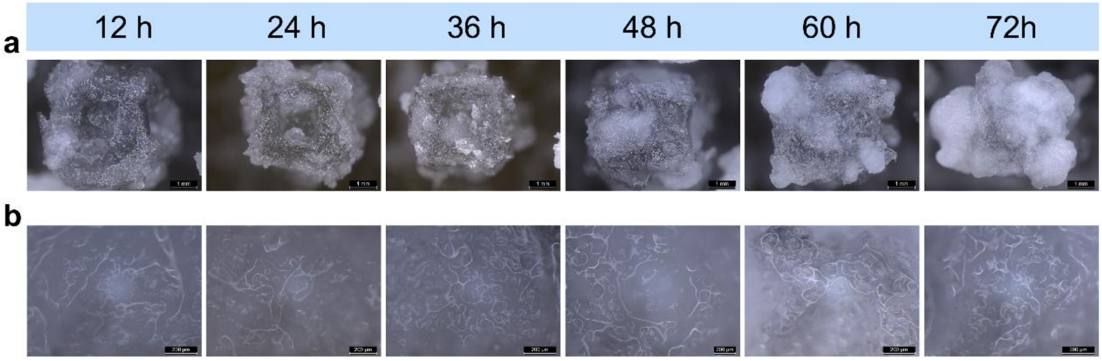

Figure S18. Microscope pictures of salt formation on ATSC under different light durations, one unit cell a) and porous wet salt b).

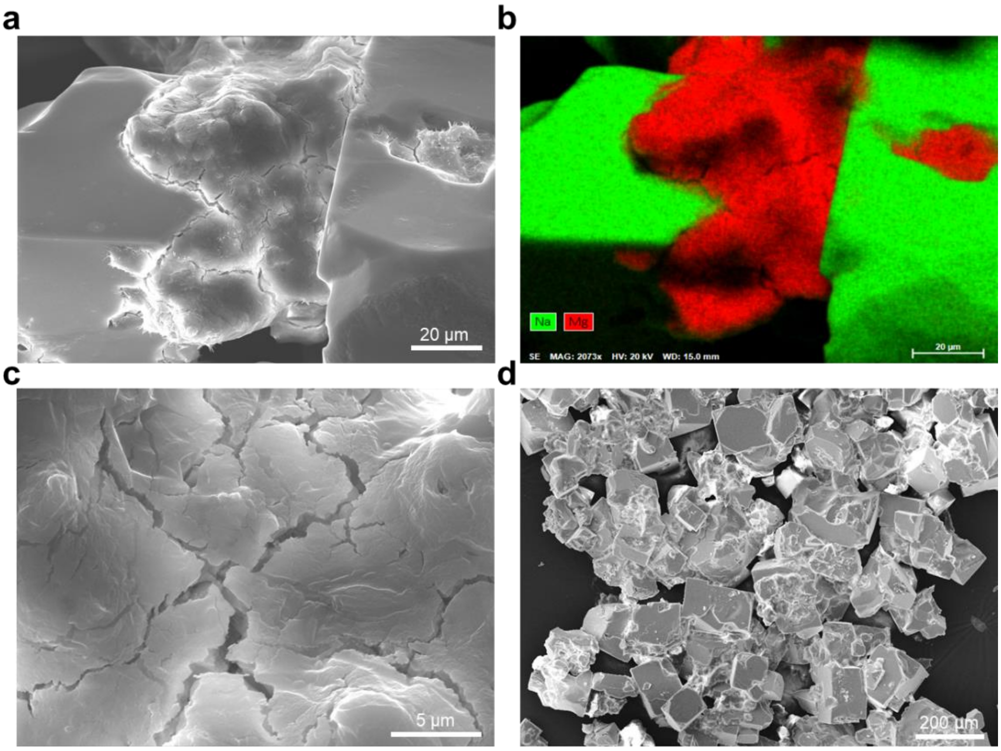

Figure S19. The morphology and elemental distribution of the salt crystals collected from ATSC when treating concentrated seawater: a) the SEM image with high magnification; b) the corresponding elemental distribution maps of a); c) the SEM image of magnesium sulfate salt crystals formed on ATSC, which have multiple cracks; d) the SEM image with low magnification.

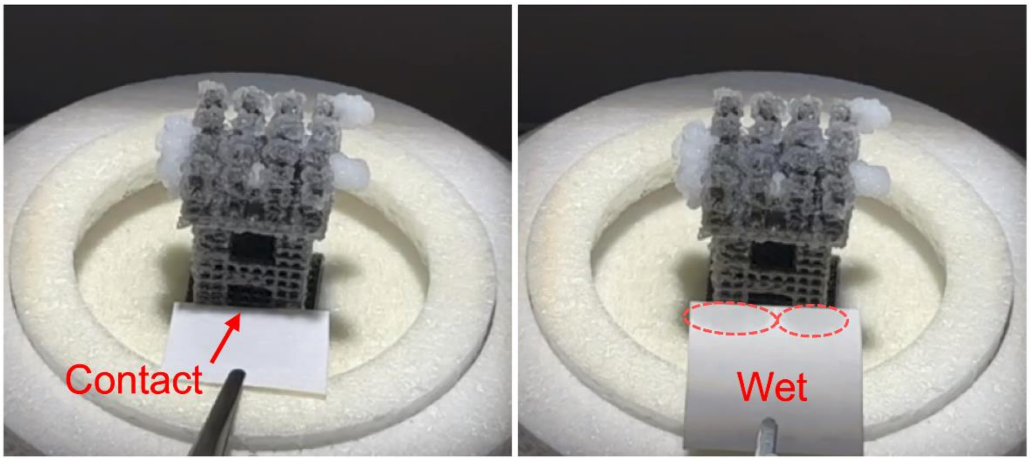

Figure S20. When a piece of filter paper was brought into contact with the salt layer on the trunk portion of ATSC, water can be absorbed from the salt, indicating that the salt layer is wet, thus the salt layer does not affect the water transport ability.

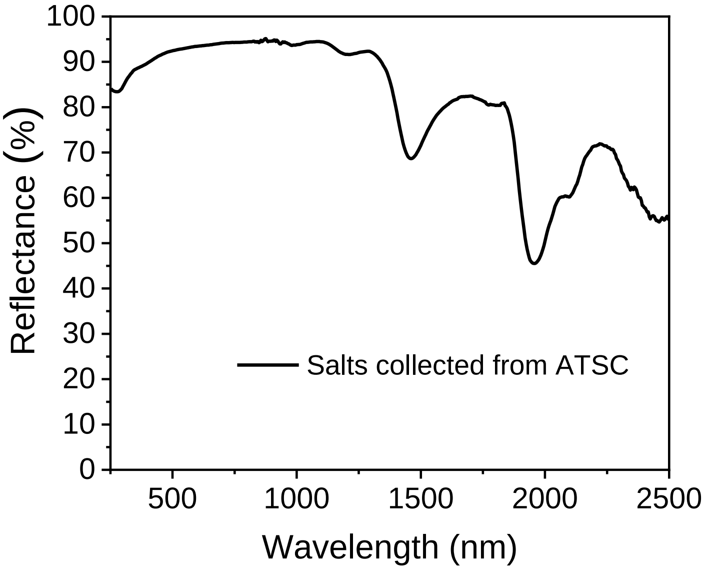

Figure S21. The solar absorption spectra (250-2500 nm) of collected salts.

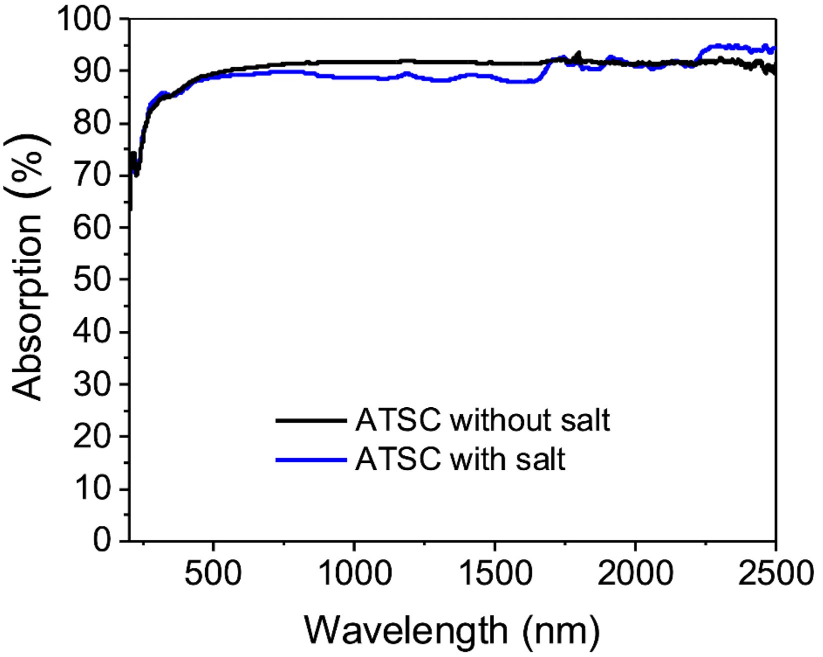

Figure S22. The solar absorption spectra (250-2500 nm) of different solar evaporators, including the ATSC without salt and the ATSC with salt.

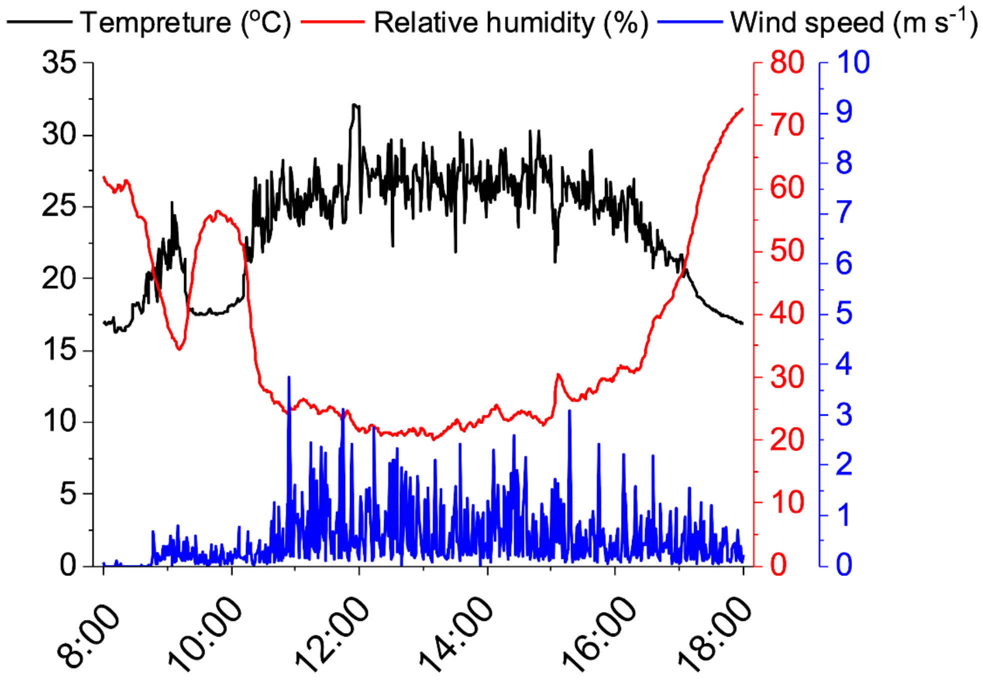

Figure S23. Real-time temperature, relative humidity, and wind speed variation of the outdoor test from 8:00 to 18:00 on December 27, 2022.

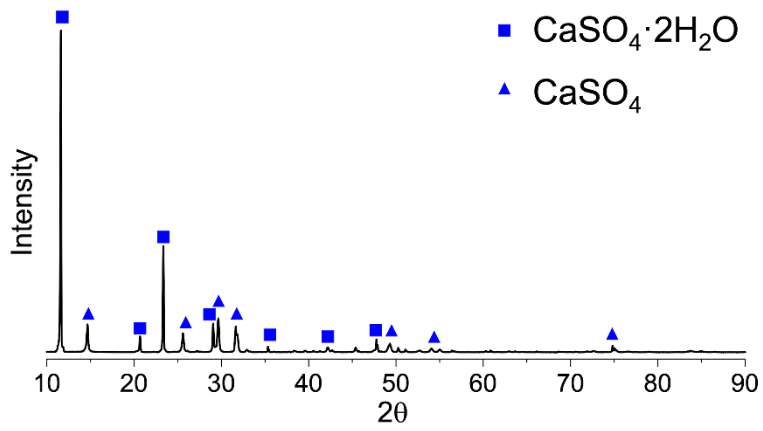

Figure S24. The X-ray diffraction (XRD) pattern of the precipitates collected during the concentration of natural seawater. Their main compositions were calcium sulfate hydrate ($CaSO_4{\cdot}2H_2O$) and calcium sulfate ($CaSO_4$).

Table S1. The ion composition of natural seawater samples.

Ion composition [g L^{-1}]	Raw seawater	Concentrated seawater
Na^+	8.8	54.0
K^+	0.5	4.0
Mg^{2+}	1.2	5.3
Ca^{2+}	0.4	1.3

Table S2. Summary of references on the evaporation performance of 3D evaporators and 3D solar crystallizers.

**Evaporator**	**Salinity**	**Evaporation rate [kg m^{-2} h^{-1}]**	**Energy efficiency [%]**	**Duration [h]**	**Ref.**
3D cup-shaped evaporator	0	2.04	140	-	[11]
	3.5 wt% NaCl	1.63	-	-	
	real seawater	1.59	-	-	
3D cylindrical solar crystallizer	0	1.70	96.7	-	[12]
	25 wt% NaCl	1.26	-	120	
3D solar crystallizer	real concentrated seawater	2.42	-	288[a)]	[13]
	0	2.09	138.5	-	
	24 wt% NaCl	1.61	-	24	
3D conical solar crystallizer	0	2.63	98.3	-	[14]
	25 wt% NaCl	2.24	97.1	-	
3D truncated conical evaporator	0	1.73	95.86	-	[15]
3D T-shaped synthetic tree crystallizer	3.5 wt% NaCl	2.03	75	60	[16]
Janus 3D solar crystallizer	0	1.66	104.1	-	[17]
	20 wt% NaCl	2.29 (1.5 sun)	-	12[a)]	
3D artificial tree evaporator	0	1.08	74	-	[18]
3D bimodal porous solar evaporator	15 wt% NaCl	6.4 (6 sun)	78	7	[19]
3D Janus wood-based evaporator	0	1.35	92.3	-	[20]
	20 wt% NaCl	1.20	82.0	-	
3D hydrogel evaporator	20 wt% NaCl	3.53	-	8 (7 days)	[2]

3D mushroom-shaped crystallizer	3.5 wt% NaCl	0.95	-	600	[21]
3D bionic evaporator	10 wt% NaCl	1.39	-	8	[22]
3D cellular solar crystallizer	0	3.64	175	-	This work
	24 wt% NaCl	2.96	150	-	
	real concentrated seawater	1.94	-	80	

Note: a) indicates solar crystallizer with additional antiscalant; - indicates no relevant content in the reference; unless otherwise specified, evaporation rate was all measured under 1 sun.

References

[1] Y. Shi, R. Y. Li, Y. Jin, S. F. Zhuo, L. Shi, J. Chang, S. Hong, K. C. Ng, P. Wang, *Joule* **2018**, *2*, 1171.

[2] X. H. Liu, F. X. Chen, Y. K. Li, H. J. Jiang, D. D. Mishra, F. Yu, Z. H. Chen, C. Q. Hu, Y. Chen, L. T. Qu, W. T. Zheng, *Adv. Mater.* **2022**, *34*, 2203137.

[3] W. Wang, Y. Shi, C. Zhang, S. Hong, L. Shi, J. Chang, R. Li, Y. Jin, C. Ong, S. Zhuo, *Nat. Commun.* **2019**, *10*, 3012.

[4] X. Y. Song, H. C. Song, N. Xu, H. F. Yang, L. Zhou, L. W. Yu, J. Zhu, J. Xu, K. J. Chen, *J. Mater. Chem. A* **2018**, *6*, 22976.

[5] N. Xu, X. Hu, W. Xu, X. Li, L. Zhou, S. Zhu, J. Zhu, *Adv. Mater.* **2017**, *29*, 1606762.

[6] X. Q. Li, J. L. Li, J. Y. Lu, N. Xu, C. L. Chen, X. Z. Min, B. Zhu, H. X. Li, L. Zhou, S. N. Zhu, T. J. Zhang, J. Zhu, *Joule* **2018**, *2*, 1331.

[7] Q. Zhang, X. F. Xiao, G. M. Zhao, H. J. Yang, H. H. Cheng, L. T. Qu, W. L. Xu, X. B. Wang, *J. Mater. Chem. A* **2021**, *9*, 10945.

[8] H. Ghasemi, G. Ni, A. M. Marconnet, J. Loomis, S. Yerci, N. Miljkovic, G. Chen, *Nat. Commun.* **2014**, *5*, 4449.

[9] Z. Wang, Q. Ye, X. Liang, J. Xu, C. Chang, C. Song, W. Shang, J. Wu, P. Tao, T. Deng, *J. Mater. Chem. A* **2017**, *5*, 16359.

[10] K. Yang, T. Pan, S. Dang, Q. Gan, Y. Han, *Nat. Commun.* **2022**, *13*, 6653.

[11] Y. Shi, R. Y. Li, Y. Jin, S. F. Zhuo, L. Shi, J. Chang, S. Hong, K. C. Ng, P. Wang, *Joule* **2018**, *2*, 1171.

[12] Y. Shi, C. L. Zhang, R. Y. Li, S. F. Zhuo, Y. Jin, L. Shi, S. Hong, J. Chang, C. S. Ong, P. Wang, *Environ. Sci. Technol.* **2018**, *52*, 11822.

[13] C. L. Zhang, Y. Shi, L. Shi, H. X. Li, R. Y. Li, S. Hong, S. F. Zhuo, T. J. Zhang, P. Wang, *Nat. Commun.* **2021**, *12*, 998.

[14] L. Wu, Z. C. Dong, Z. R. Cai, T. Ganapathy, N. X. Fang, C. X. Li, C. L. Yu, Y. Zhang, Y. L. Song, *Nat. Commun.* **2020**, *11*, 521.

[15] S. H. Sun, C. C. Shi, Y. D. Kuang, M. S. Li, S. Li, H. F. Chan, S. K. Zhang, G. X. Chen, A. Nilghaz, R. Cao, J. F. Tian, *Water Res.* **2022**, *226*, 119279.

[16] Y. Shao, J. Tang, N. Li, T. Sun, L. Yang, D. Chen, H. Zhi, D. Wang, H. Liu, G. Xue, *EcoMat* **2020**, *2*, e12018.

[17] R. Gu, Z. Yu, Y. Sun, Y. Su, W. Wu, S. Cheng, *Desalination* **2022**, *537*, 115862.

[18] H. Liu, C. J. Chen, G. Chen, Y. D. Kuang, X. P. Zhao, J. W. Song, C. Jia, X. Xu, E. Hitz, H. Xie, S. Wang, F. Jiang, T. Li, Y. J. Li, A. Gong, R. G. Yang, S. Das, L. B. Hu, *Adv. Energy Mater.* **2018**, *8*, 1701616.

[19] S. M. He, C. J. Chen, Y. D. Kuang, R. Y. Mi, Y. Liu, Y. Pei, W. Q. Kong, W. T. Gan, H. Xie, E. Hitz, C. Jia, X. Chen, A. Gong, J. M. Liao, J. Li, Z. J. Ren, B. Yang, S. Das, L. B. Hu, *Energy Environ. Sci.* **2019**, *12*, 1558.

[20] X. Chen, S. M. He, M. M. Falinski, Y. X. Wang, T. Li, S. X. Zheng, D. Y. Sun, J. Q. Dai, Y. H. Bian, X. B. Zhu, J. Y. Jiang, L. B. Hu, Z. J. Ren, *Energy Environ. Sci.* **2021**, *14*, 5347.

[21] Y. Xia, Q. F. Hou, H. Jubaer, Y. Li, Y. Kang, S. Yuan, H. Y. Liu, M. W. Woo, L. Zhang, L. Gao, H. T. Wang, X. W. Zhang, *Energy Environ. Sci.* **2019**, *12*, 1840.

[22] N. Xu, J. L. Li, Y. Wang, C. Fang, X. Q. Li, Y. X. Wang, L. Zhou, B. Zhu, Z. Wu, S. N. Zhu, J. Zhu, *Sci. Adv.* **2019**, *5*, eaaw7013.
